# Folic Acid-Functionalized Chitosan-Coated Oleosomes Engineered for Enhanced Anti-Arthritic Delivery of *Syzygium cumini* Seed Extract

**DOI:** 10.3390/ph19101658

**Published:** 2026-10-20

**Authors:** Samar M. Aborhyem, Neveen Agamy, Aamena F. Abdallah, Shimaa O. Ali, Haidy Abbas, Mariam Zewail

**Affiliations:** 1 Nutrition Department, High Institute of Public Health, Alexandria University, Alexandria 21561, Egypt; hiph.samaraborhyem@alexu.edu.eg (S.M.A.); neveenagamy@alexu.edu.eg (N.A.); hiph.ammnahfathullah@alexu.edu.eg (A.F.A.); 2 Human Nutrition and Dietetics, College of Health Sciences, Abu Dhabi University, Abu Dhabi P.O. Box 59911, United Arab Emirates; 3 Biochemistry Department, Faculty of Pharmacy, Cairo University, Kasr el Ainy St., Cairo 11562, Egypt; shimaa.omar@pharma.cu.edu.eg; 4 Department of Pharmaceutics, Faculty of Pharmacy, Damanhour University, Damanhour 22511, Egypt; haidy.abass@pharm.dmu.edu.eg

**Keywords:** *Syzygium cumini*, folic acid functionalization, autoimmune diseases, inflammation, oleosomes

## Abstract

**Background/Objectives**: This study evaluated the therapeutic efficacy of folic acid-functionalized chitosan-coated oleosomes (F-CS-OL) loaded with *Syzygium cumini* (SYG) seed extract against rheumatoid arthritis (RA) in a complete Freund’s adjuvant (CFA)-induced arthritis rat model. **Methods**: Rats received treatments via the oral route for 11 days following arthritis induction. The colloidal formulation exhibited an average particle size of 80.78 ± 1.02 nm, a zeta potential of −15.5 ± 1.12 mV, and an encapsulation efficiency (EE) of 94.98 ± 0.56%, alongside characterization by TEM, FTIR, and DSC. CFA-induced arthritis produced a pronounced inflammatory and angiogenic response, marked by significantly elevated serum levels of TNF-α, IL-6, CRP, VEGF, ICAM-1, MMP-3, and CXCL13 compared with the normal control group. **Results**: While treatment with free SYG extract resulted in moderate reductions in these biomarkers, the nanoencapsulated extract (SYG-loaded F-CS-OL) produced significantly greater reductions in inflammatory, chemotactic, matrix-remodeling, and angiogenesis-associated biomarkers compared with the untreated CFA-induced arthritis group. Histopathological examination of joint tissues further supported the biochemical findings. **Conclusions**: Overall, SYG-loaded F-CS-OL exhibited potent anti-inflammatory, immunomodulatory activities and significantly reduced biomarkers associated with angiogenic signaling in CFA-induced arthritis in CFA-induced arthritis. These findings suggest that folic acid-functionalized nanocarriers may represent a promising strategy for enhancing the therapeutic potential of SYG; however, further pharmacokinetic and safety studies are warranted to support clinical translation.

## 1. Introduction

Rheumatoid arthritis (RA) is a chronic systemic autoimmune disorder marked by severe and progressive symmetric polyarthritis. A pathological characteristic of RA is the abnormal hyperplasia of the synovial membrane. The inflamed tissue displays a vigorous inflammatory response, marked by significant activation of fibroblasts and macrophages, infiltration of innate and adaptive immune cells, angiogenesis, and the formation of pannus [1].

In an inflamed synovium, fibroblast-like synoviocytes (FLS) release chemokines, proinflammatory factors like interleukin-6 (IL-6) and tumor necrosis factor-alpha (TNF-α), and matrix metalloproteinases (MMPs) that degrade cartilage and extracellular matrix (ECM) [2,3]. RA synovial fibroblasts (RASFs) secrete proinflammatory mediators, which attract more immune cells to infiltrate the synovium and promote synovial inflammation [4]. The synovial lining layer’s proinflammatory cytokines modulate adhesion molecules, MMPs, chemokines, and growth factors, regulating cell–cell signaling throughout the angiogenic stages [5].

Angiogenesis plays a pivotal role in the pathogenesis and progression of RA, particularly in synovial tissue expansion, which facilitates leukocyte recruitment, synovial hyperplasia, and the formation of invasive pannus tissue [6]. This process is initiated by pro-angiogenic factors, such as vascular endothelial growth factor (VEGF), which bind to receptors on endothelial cells and promote endothelial cell activation, proliferation, and neovascularization. In addition to angiogenesis, activated macrophages are key contributors to RA progression. These cells are recruited from the circulation to inflamed synovial tissues through a multistep process involving endothelial adhesion, transmigration (extravasation), and chemokine-guided migration [7]. Upon infiltration into the synovium, macrophages accumulate within the inflamed pannus and produce a broad range of pro-inflammatory cytokines, chemokines, and matrix-degrading enzymes that perpetuate joint inflammation and destruction. The recruitment and retention of macrophages are facilitated by adhesion molecules, including intercellular adhesion molecule-1 (ICAM-1), which mediate leukocyte–endothelial interactions and migration into inflamed tissues [7].

Chemokines are also involved in a variety of pathophysiological processes in RA; they play an important role in homeostasis, cellular immune activity synthesis, and pathological immune contributions to various diseases [8]. Inflammation is associated with C-X-C motif chemokine ligand 13 (CXCL13), a well-known indicator of RA severity. Unlike other chemokine receptors, CXCL13 only works as a chemotactic agent when it interacts with CXCR5, indicating CXCR5’s particular reaction to CXCL13 [9].

Substantial advances in RA management have been achieved through the use of conventional synthetic DMARDs, biologic therapies, and Janus kinase (JAK) inhibitors, which can reduce disease activity, improve functional outcomes, and slow structural joint damage [10,11]. Nevertheless, a proportion of patients continue to experience incomplete responses, disease relapse, treatment-associated adverse effects, or progressive disease despite optimized therapy [12]. These limitations have stimulated interest in alternative and adjunctive therapeutic approaches, including bioactive natural products and targeted drug-delivery systems, which may offer complementary anti-inflammatory effects while potentially reducing systemic exposure and off-target toxicity.

One of the common medicinal plants used to cure a range of illnesses is *Syzygium cumini* (S. cumini) (L.) Skeels (jambolan) (SYG) [13]. Due to bioactive substances including phenols, flavonoids, and tannins found in different plant sections, SYG exhibits a variety of pharmacological characteristics, including antidiabetic, anti-inflammatory, antibacterial, antioxidant, cardioprotective, and hepatoprotective effects [13,14].

Despite the therapeutic promise of medicinal plants like SYG, the clinical application of natural products is hindered by multiple physicochemical and biopharmaceutical limitations such as inadequate solubility, stability, limited bioavailability, extensive first-pass metabolism, brief circulation half-life and substantial binding to plasma proteins [15,16].

The nano formulation approach provides an effective solution to these challenges by enhancing drug solubility, protecting bioactive compounds from premature degradation, increasing absorption across biological membranes, enabling targeted delivery to specific tissues, prolonging systemic circulation time and ultimately improving the therapeutic efficacy of natural phytochemicals. Phospholipid-based nano-delivery systems have been investigated to improve drug transport characteristics such as intestinal permeability, highlighting that lipid association can significantly enhance drug bioavailability [17]. Moreover, the inclusion of oleic acid into phospholipid vesicles to prepare oleosomes enhances the stability of the vesicles and promotes their interaction with biological membranes, in addition to its effects as an anti-inflammatory [15,18].

Modifications of phospholipid vesicles have been reported with a variety of polymers, including chitosan (CS), which is a naturally occurring, biocompatible polymer endowed with multiple intrinsic properties that facilitate joint healing. It also exhibits immunomodulatory activities, such as the ability to suppress pro-inflammatory cytokine production and the stimulation of chondrocyte proliferation [19]. CS is regarded as one of the most suitable coating polysaccharides for lipid nanocarriers. This preference stems from its inherent positive surface charge, which readily interacts with the negatively charged surfaces of lipid nanocarriers, enabling effective electrostatic binding [19].

Activated macrophages play a central role in the pathogenesis of RA through the production of pro-inflammatory cytokines, chemokines, and tissue-destructive mediators. Among the surface markers expressed by these cells, folate receptor-β has emerged as a particularly attractive target because of its selective upregulation on activated macrophages within inflamed tissues and its limited expression in normal tissues. Consequently, folate-mediated targeting strategies have been widely investigated for both imaging and therapeutic applications in RA, demonstrating the potential of folic acid-conjugated systems to selectively interact with activated macrophages and improve site-specific delivery to inflamed joints [20,21,22,23].

Despite the well-documented antioxidant, anti-inflammatory, and immunomodulatory properties of SYG seed extract, its therapeutic potential in RA remains insufficiently explored. Although folic acid- and chitosan-functionalized lipid nanocarriers have previously been investigated by our group using solid lipid nanoparticle platforms [19], the current study explores a distinct phospholipid-based oleosomal system incorporating oleic acid, CS surface coating, and folic acid functionalization for delivery of SYG seed extract. Therefore, a significant research gap exists regarding the ability of a targeted nano-delivery platform to enhance the anti-rheumatic efficacy of SYG. Based on the overexpression of folate receptors on activated macrophages within inflamed joints, we hypothesized that encapsulating SYG within folic acid-functionalized, chitosan-coated oleosomes would enhance its accumulation at inflammatory sites and consequently improve its anti-inflammatory, anti-angiogenic, and immunomodulatory effects compared with the free extract. Accordingly, the present study aimed to develop and characterize SYG-loaded oleosomes coated layer-by-layer with chitosan and folic acid, representing, to the best of our knowledge, the first report of SYG-loaded folic acid-functionalized chitosan-coated oleosomes evaluated in CFA-induced arthritis.

## 2. Results and Discussion

### 2.1. SYG Seed Extract Characterization

**Figure 1:**
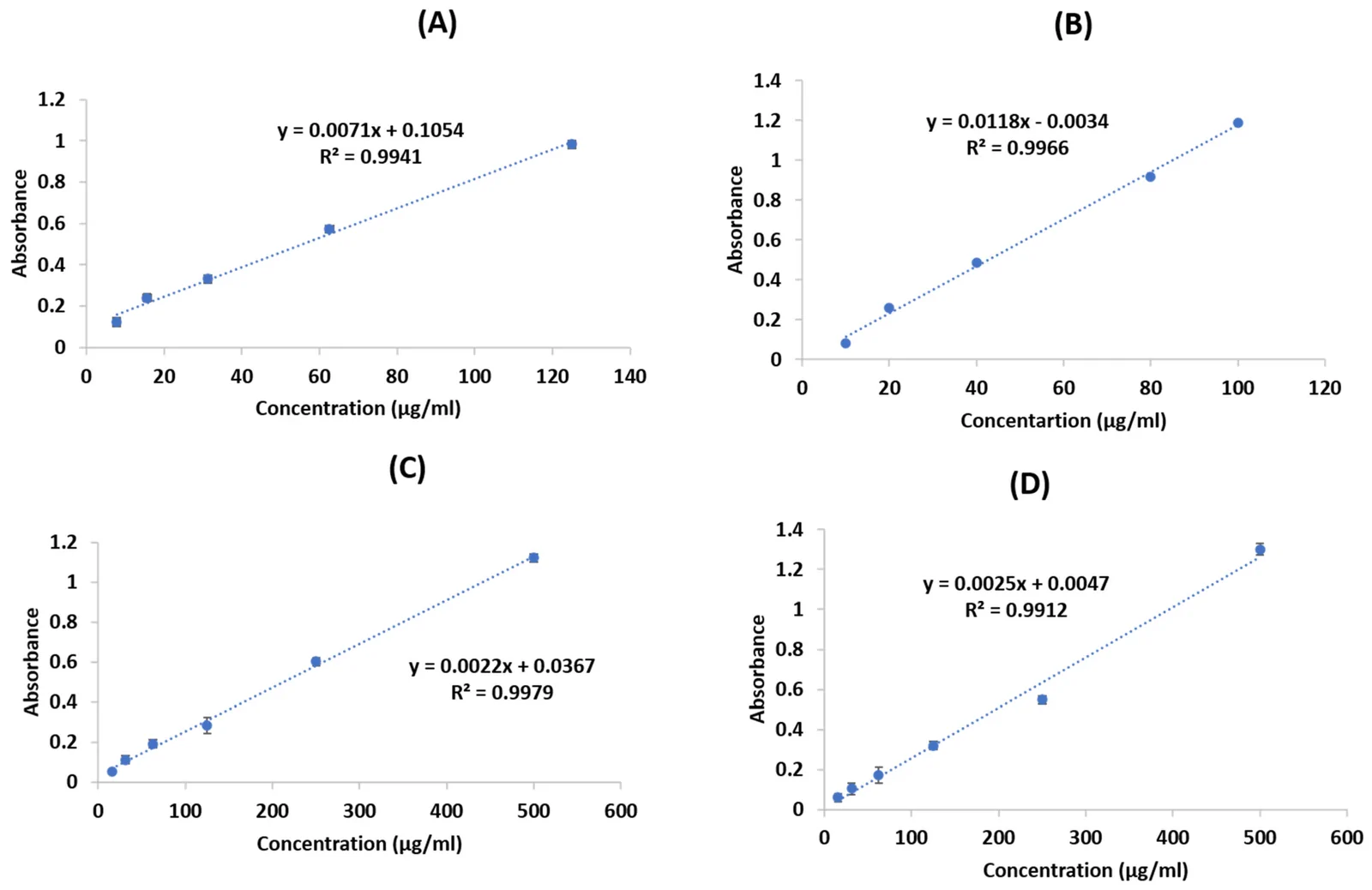
Calibration curves of phytochemical standards: (**A**) Escin standard, (**B**) tannic acid standard, (**C**) phenolic standard (gallic acid standard), and (**D**) flavonoid standard (quercetin standard).

The phytochemical composition of the SYG seed extract is summarized in Table 1, while Figure 1A–D presents the corresponding calibration curves used for quantification. The antioxidant activity of the extract was evaluated separately using the relevant antioxidant assays. The SYG seed extract contains relatively high Vanillin-reactive saponin content, with a mean of 10.38 ± 0.07 mg escin-equivalents per gram of dry matter. The very small spread among replicates indicates tight experimental reproducibility. The vanillin-sulfuric acid assay suggested the presence of compounds with saponin-like reactivity. However, the assay is not entirely specific, and the obtained values should be interpreted as saponin-equivalent estimates rather than definitive quantification of individual saponins. Saponins are associated with surface-active properties and potential biological activities such as anti-inflammatory, antimicrobial, and gastroprotective effects [24,25,26]. The magnitude reported here is consistent with saponin-rich fractions described in Jamun seed extracts in prior work, which frequently enumerate saponin content in the mg/g range and show variability with solvent and part of the plant [25]. The tannin content is high, with a mean around 35.53 mg tannic acid equivalents per ml. Tannins are frequently reported in SYG seeds and related parts and are cited as contributing to antioxidant, astringent, and antimicrobial properties in various Jamun-derived preparations [26]. The high tannin level here aligns with the understanding that seed-related extracts can be tannin-rich, contributing to radical scavenging and potential enzyme-inhibition activities in pharmacological contexts.

Total phenolic content was 40.5 mg GAE/gm dry extract. They are a broad proxy for antioxidant capacity and are consistently reported in SYG spp. literature as a driver of DPPH scavenging and related antioxidant measures [27].

The flavonoid content was 30.5 mg quercetin equivalents (QE) per gm dry extract. Flavonoids represent a major subclass of phenolic compounds and are widely associated with antioxidant, anti-inflammatory, and enzyme inhibitory activities in materials derived from SYG [24,25]. The relatively high flavonoid concentration observed in the SYG seed extract suggests the presence of significant bioactive potential.

Moreover, the concurrent presence of saponins, tannins, phenolics, and flavonoids indicates a diverse phytochemical profile that may contribute to multiple biological activities commonly reported for SYG derivatives. These include antioxidant, antimicrobial, anti-inflammatory, and potential antidiabetic effects, which are likely mediated through the redox properties of phenolic and flavonoid compounds as well as enzyme-modulating activities associated with tannins that provide a solid basis for pursuing targeted bioactivity assays and formulation considerations in future work [26].

**Figure 2:**
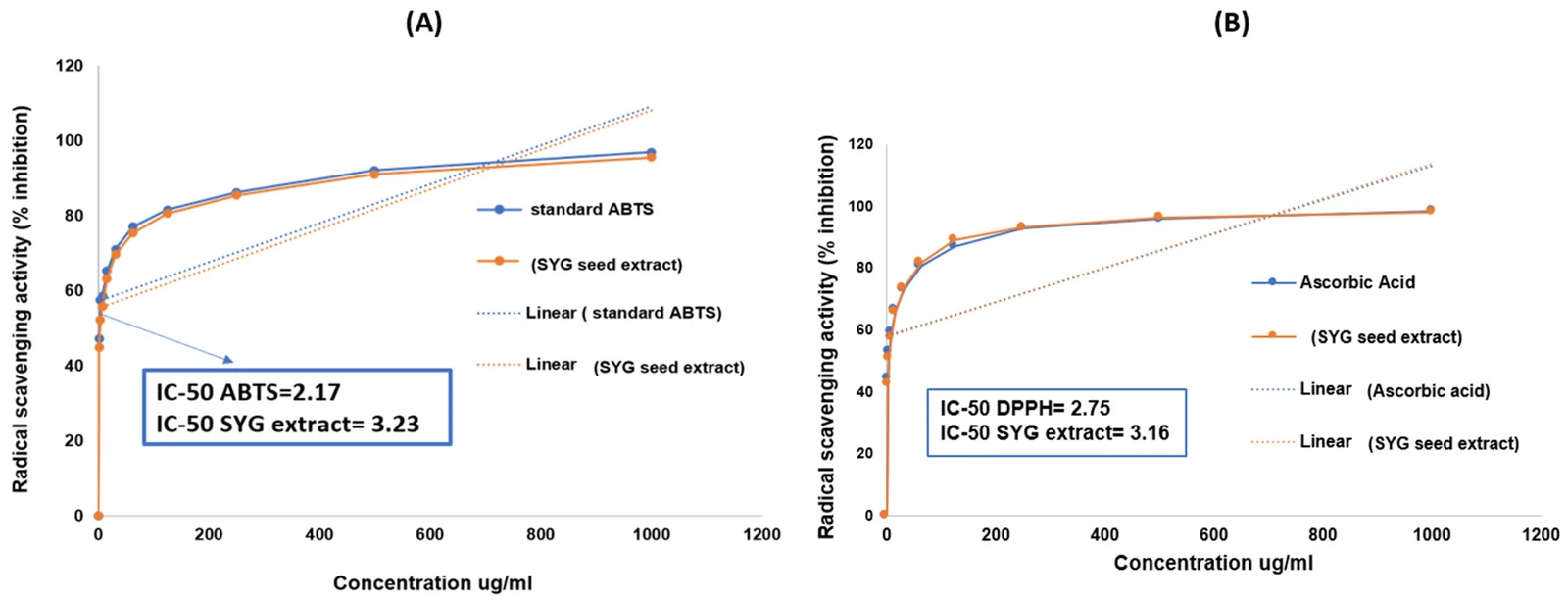
(**A**) ABTS radical-scavenging activity of SYG seed extract compared with Trolox reference standard. (**B**) DPPH Radical scavenging activity of SYG seed extract compared with ascorbic acid reference standard. Values are presented as mean ± SE (*n* = 3). Concentration range: 1.95–1000 µg/mL.

The antioxidant activity of the SYG seed extract was evaluated using two in vitro radical-scavenging assays, namely ABTS and DPPH, with results compared against established reference antioxidants. The extract exhibited notable radical-scavenging activity, with IC_50_ values of 3.23 µg/mL and 3.16 µg/mL in the ABTS and DPPH assays, respectively (Figure 2). For comparison, Trolox and ascorbic acid exhibited IC_50_ values of 2.17 µg/mL and 2.75 µg/mL in the corresponding assays. These findings indicate that the SYG seed extract possesses measurable free radical-scavenging activity under the experimental conditions employed and are consistent with previous reports describing the antioxidant potential of *SYG* seeds [28].

Phenolic and flavonoid compounds are widely recognized for their capacity to act as hydrogen or electron donors, thereby contributing to free-radical stabilization and interruption of oxidative chain reactions [29]. The observed antioxidant activity may be associated with the overall phytochemical composition of the extract, which contained saponin-equivalent compounds (10.4 mg AE/g), tannins (35.5 mg TAE/g dry extract), total phenolics (40.5 mg GAE/g dry extract), and total flavonoids (30.5 mg QE/g dry extract). However, the present assays do not identify the specific constituents responsible for the observed activity, and the relative contribution of individual compounds was not investigated in this study. Furthermore, DPPH and ABTS assays provide only a preliminary assessment of radical-scavenging activity and do not fully represent the complex oxidative processes involved in rheumatoid arthritis pathogenesis. Therefore, further phytochemical characterization and mechanistic studies are required to identify the active constituents and elucidate the pathways underlying the antioxidant effects of the extract. Future studies should evaluate hydroxyl radical scavenging, superoxide scavenging, metal-chelating activity, and cellular oxidative stress markers to provide a more comprehensive assessment of its antioxidant potential.

### 2.2. Preparation and Characterization of SYG-Loaded F-CS-OL

#### 2.2.1. Particle Size, PDI and Zeta Potential

**Figure 3:**
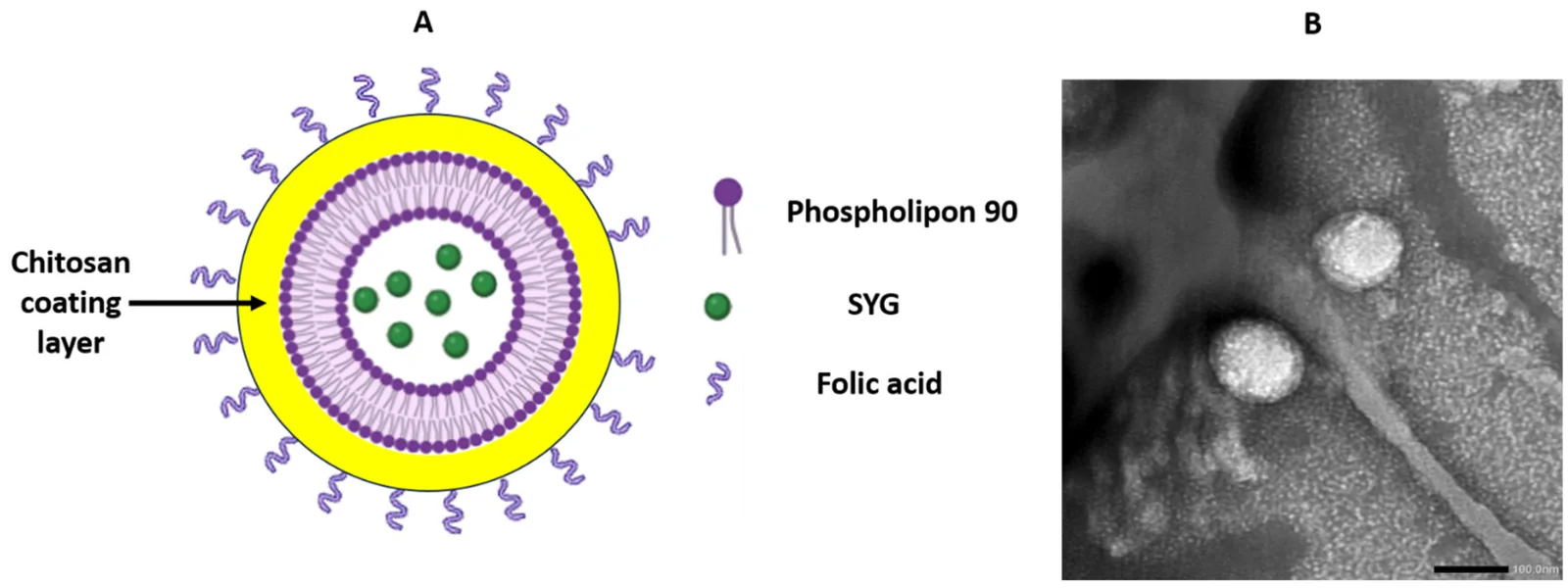
(**A**) Schematic diagram of SYG-loaded F-CS-OL and (**B**) TEM micrograph of SYG-loaded F-CS-OL (F7).

The thin film hydration technique is one of the simplest methods for the preparation of phospholipid vesicles. It allows the encapsulation of both hydrophilic and lipophilic drugs [30]. The choice of phospholipid-based vesicles is based on their biocompatible amphiphilic nature, making them an excellent excipient for nanoformulations, facilitating enhanced therapeutic applications in drug delivery systems [31]. Oleic acid in oleosomes has been shown to enhance liposome stability and deformability as well as to have intrinsic anti-inflammatory, antioxidant, and antibacterial qualities that may increase the therapeutic efficacy of some medications [32]. Tween 80 was incorporated in the formulation as a permeation enhancer, as it confers vesicle deformation [17]. Protonated CS amino groups and negatively charged phospholipid headgroups interact electrostatically to form a CS coating, which can reduce bilayer permeability and impede the diffusion of encapsulated medication out of the carrier [17]. Folic acid was selected as an active targeting ligand that can bind to folic acid receptors that are overexpressed on macrophages in RA [19]. Figure 3A represents a schematic diagram of SYG-loaded F-CS-OL. Cholesterol (2 mg) and Tween 80 (40 mg) were included at fixed concentrations in all formulations and were therefore not originally listed as variable formulation factors. Cholesterol was incorporated at a low concentration as a membrane-stabilizing excipient; however, its proportion relative to phospholipid was minimal, and therefore its contribution to vesicle physicochemical properties is expected to be limited compared with those of oleic acid and Tween 80.

All reported particle size, PDI, zeta potential, and EE measurements were performed on freshly prepared and purified vesicle dispersions after washing to remove unencapsulated SYG and excess coating materials, and prior to freeze-drying.

The effects of oleic acid, SYG incorporation, CS and folic acid coating on the colloidal characteristics of vesicles were investigated. It can be noted that the incorporation of oleic acid significantly (One-way ANOVA, *p* < 0.0001) reduced the particle size of liposomes from 54.39 ± 0.56 (F1) to 47.41± 0.87 nm (F2). This aligns with the previous results that demonstrate the effect of oleic acid inclusion in phospholipid vesicles on reducing the particle size of the resultant vesicles due to its effect on membrane fluidity [33]. The inclusion of SYG in F2 significantly increased its particle size to 54.42 ± 0.34 nm (F3). The same effect was noted upon loading F4 and F6 with SYG to prepare F5 and F7, respectively. These findings are consistent with the previous report by Makhlof et al., who reported that the particle size of CS coated OL increased significantly upon loading them with quercetin [17]. Moreover, from Table 2, it can be noted that coating OL with CS increased their particle size from 54.42 ± 0.34 (F3) to 66.80 ± 0.98 (F5). This is consistent with previous reports about the effect of coating lipid nanocarriers with CS on the particle size of the resultant vesicles [19,34].

All formulations exhibited PDI values less than 0.3, indicating the homogeneity of the prepared nanoformulations. F2 and F3 demonstrated the smallest PDI value around 0.180; on the other hand, F6 had the highest PDI value (0.263 ± 0.013). All formulations exhibited PDI values below 0.3, indicating relatively narrow particle size distributions and acceptable colloidal uniformity. However, these values should not be interpreted as evidence of complete homogeneity. A more pronounced increase in particle size was observed after folic acid coating of the drug-loaded vesicles (F5 to F7) compared with the blank vesicles (F4 to F6). The increase in particle size observed for F7 compared with F5 could be attributed to the deposition of the folic acid-containing coating layer onto the vesicle surface, as evidenced by the concomitant reversal in zeta potential from +17.25 ± 0.56 mV to −15.5 ± 1.12 mV, as it will be discussed later. Similar increases in particle size following chitosan- and folate-based surface functionalization have been reported previously [17,35]. Liposomes and oleosomes exhibited negative zeta potential values attributable to the phospholipid head groups and the ionized carboxyl groups of oleic acid. Incorporation of oleic acid increased the magnitude of the negative surface charge from −16.2 ± 1.44 mV (F1) to −20.6 ± 1.32 mV (F2) [17].

Upon loading SYG, the zeta potential shifted to −15.1 ± 0.98 mV (F3), which may be attributed to interactions between extract constituents and the vesicle surface, leading to partial shielding of negatively charged groups. CS coating resulted in charge inversion to the positive side (F4, +16.7 ± 2.1 mV; F5, +17.25 ± 0.56 mV) due to electrostatic interactions between the positively charged amino groups of CS and the negatively charged phospholipid surface [36,37,38,39]. Subsequent folic acid coating reversed the surface charge to the negative range (F6, −14.7 ± 1.89 mV; F7, −15.5 ± 1.12 mV), consistent with the presence of negatively charged folate moieties on the particle surface [40]. This finding coincides with the previous report by Zewail et al., who reported that coating leflunomide-loaded solid lipid nanoparticles inverted their surface charge to the positive side and subsequent coating with folic acid resulted in negatively charged particle surfaces [40]. However, zeta potential measurements alone cannot conclusively differentiate surface-bound folic acid from folate-chitosan complexes or residual associated folic acid and should therefore be interpreted in conjunction with the particle size and FTIR results.

The final formulation (F7) was selected prospectively based on predefined criteria including high encapsulation efficiency, nanometric particle size, low PDI (<0.3), appropriate surface charge, and successful folic acid functionalization. Although folic acid coating increased particle size relative to F5, F7 exhibited the highest encapsulation efficiency (94.98 ± 0.56%), maintained acceptable colloidal characteristics (particle size <100 nm and PDI <0.3), and provided the active targeting functionality required for folate receptor-mediated macrophage uptake. Therefore, F7 was selected as the optimal formulation for subsequent investigations.

#### 2.2.2. TEM

As demonstrated in Figure 3B, SYG-loaded F-CS-OL appeared as spherical, uniform particles with no signs of aggregation or clustering.

#### 2.2.3. EE% and DL%

**Figure 4:**
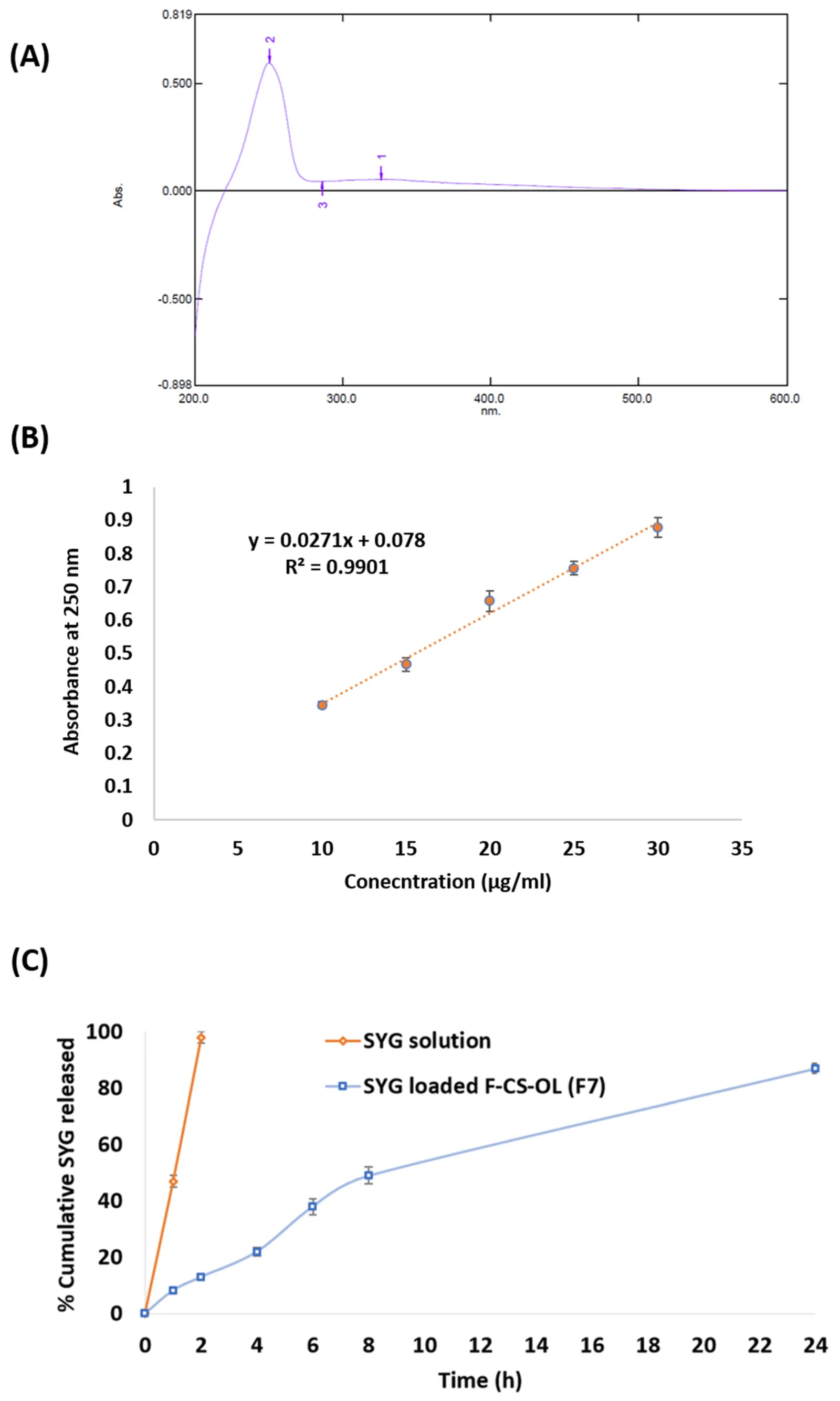
(**A**) UV spectrum of SYG demonstrating characteristic peaks, (**B**) calibration curve of SYG at 250 nm and (**C**) in vitro drug release in PBS in pH 7.4, where results are represented as mean ± SEM (*n* = 3).

The UV-Vis scanning of the herbal extract SYG revealed a distinct maximum absorbance wavelength at 250 nm (Figure 4A), which was utilized for all subsequent quantification. A robust linear relationship was established for the drug concentration against absorbance, yielding the regression equation y = 0.0271x + 0.078, with R^2^ = 0.9901. UV spectra for different SYG concentrations used for the construction of the calibration curve are listed in Figure S1. Encapsulation efficiency was determined only for SYG-loaded formulations (F3, F5, and F7). OL (F3), CS-OL (F5) and F-CS-OL (F7) demonstrated high EE% (Table 2); their EE% values were 91.45 ± 1.2, 94.78 ± 0.92 and 94.98 ± 0.56, respectively. The high EE% of SYG-loaded OL is consistent with previous studies that reported the ability of OL to encapsulate both hydrophilic and lipophilic moieties with high EE% [41,42]. The increase in EE% upon coating with CS may be ascribed to the entrapment of SYG in the CS coating layer through the formation of an adsorbed, positively charged polymer layer on the particle surface that stabilizes the membrane and decreases drug leakage [17]. Also, similar effects were reported in different types of lipid-based nanoparticles upon coating with CS [19,43,44,45,46].

Although a slight increase in EE% was observed following chitosan and folic acid coating, these findings are interpreted as improved retention of SYG within the vesicles due to membrane stabilization and reduced drug leakage. Since SYG is a complex herbal extract and quantification was performed using UV spectrophotometry at 250 nm rather than a marker-specific chromatographic assay, the observed differences in EE% should be interpreted cautiously and not as conclusive evidence of additional encapsulation of specific phytochemical constituents. Future investigations should employ validated HPLC or UPLC methods targeting characteristic SYG phytochemical markers to achieve more specific assessment of encapsulation, loading, stability, and release behavior.

The DL% percentages of F3, F5, and F7 were 15.59 ± 0.20%, 16.16 ± 0.16%, and 16.19 ± 0.10%, respectively. The slight increase in drug loading after chitosan and folic acid coating was consistent with the observed increase in encapsulation efficiency (Table 2).

#### 2.2.4. In Vitro SYG Release

Release quantification was performed using UV spectrophotometry at 250 nm and therefore reflects the collective release of UV-absorbing constituents present in the SYG extract. Consequently, the reported release profile represents the overall release behavior of the extract rather than that of individual phytochemical markers. The method does not permit identification of the released constituents or assessment of their chemical integrity during release.

The in vitro release study was conducted only in PBS (pH 7.4), and therefore the influence of gastrointestinal pH conditions on formulation behavior was not evaluated. Since the formulation is intended for oral administration, assessment of release and stability under simulated gastric fluid (pH 1.2) and simulated intestinal fluid (pH 6.8) conditions would provide important information regarding the integrity of the chitosan coating, vesicle stability, and site-specific release characteristics. These investigations should be addressed in future studies.

In the dissolution studies (Figure 4C), the free SYG solution exhibited a rapid “burst” release, reaching nearly 100% within the first 2 h. In contrast, the SYG-loaded F-CS-OL nanocarriers (F7) exhibited a significantly controlled and biphasic release pattern. An initial release of approximately 13% was observed at 2 h, followed by a sustained release phase reaching 87.2% after 24 h, suggesting sustained association of SYG with the F-CS-OL system and slower release under the employed experimental conditions. These findings are along with previous reports on the ability of OL [42] and CS-OL [17] to provide sustained release profile for the loaded drugs whether hydrophilic like berberine [42] or lipophilic quercetin [17] and sildenafil [41]. The release profile observed at pH 7.4 demonstrates sustained release under the tested experimental conditions; however, it should not be assumed to represent release behavior throughout the gastrointestinal tract.

The release profiles reported herein were obtained using a dialysis-based method and therefore reflect both release from the nanocarrier and diffusion across the dialysis membrane. Although the substantially slower release observed for F-CS-OL compared with the free extract is consistent with sustained association of SYG with the vesicular system, the contribution of membrane diffusion resistance cannot be completely excluded. Consequently, the release data should be interpreted as comparative rather than absolute measures of release behavior. Future investigations using reverse dialysis, direct-dispersion methods, or sample-and-separate approaches would provide a more rigorous assessment of intrinsic release kinetics.

#### 2.2.5. FTIR

**Figure 5:**
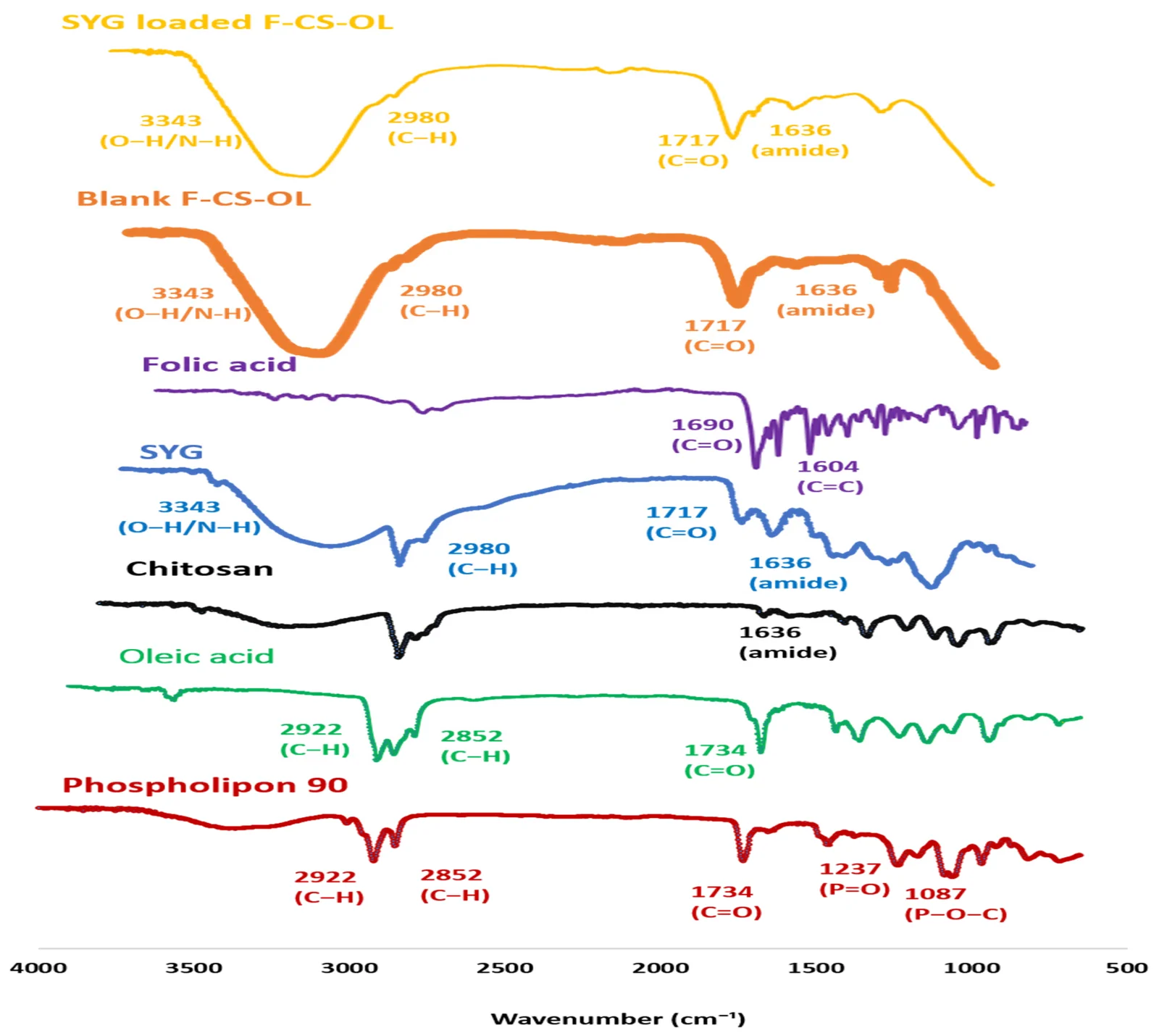
FTIR spectra of Phospholipon^®^ 90H, oleic acid, SYG, folic acid, blank F-CS-OL, and SYG-loaded F-CS-OL. Spectra are presented as transmittance (%) versus wavenumber (cm^−1^). Characteristic absorption bands discussed in the text are indicated on the spectra.

In Figure 5, SYG spectrum showed C–H stretching at 2980 cm, the peak at 1717 cm^−1^ corresponds to C=O stretching. C–H bending peak appeared at 1340 cm^−1^, meanwhile C–O stretching was noted at 1230 cm^−1^. At 1020 and 1157 cm^−1^ multiple peaks of C–O–C pyranose ring were evident in SYG spectrum [47].

O-H, amide, and C-O groups are represented by distinctive peaks in the CS spectra at 3343, 1636, and 1375 cm^−1^. The N-H and O-H stretching bands are represented by a strong band in the 2869–3544 cm^−1^ region [38].

The distinctive C–H stretching signal found in the lengthy fatty acid chain was visible in the FTIR spectrum of Phospholipon^®^ 90H at 2922 and 2852 cm^−1^, respectively. The spectra also showed a C=O stretching band at 1734 cm^−1^ in the fatty acid ester, a P=O stretching band at 1237 cm^−1^, a P–O–C stretching band at 1087 cm^−1^, and a −N+(CH3)3 stretching at 968 cm^−1^ [48].

The folic acid spectra displayed a number of distinctive peaks, such as the stretching vibrations of C=C and C=O, which were detected at 1604 and 1690 cm^−1^, respectively. The symmetrical CH3 deformation mode was represented by a strong peak at 1412 cm^−1^. N-H and O-H vibrations were attributed to the broadband between 3579 and 3317 cm^−1^, respectively [19].

The reduced intensity or apparent disappearance of the folic acid C=O stretching band in the spectrum of SYG-loaded F-CS-OL, together with changes observed in the amide region, suggests interactions between folic acid and chitosan. Because the band at 1636 cm^−1^ is characteristic of chitosan, these spectral changes are more appropriately interpreted as evidence of molecular interactions rather than the formation of new covalent bonds. Furthermore, the reduced visibility of the folic acid C=O band may result from dilution effects, spectral overlap, and the relatively low proportion of folic acid within the multicomponent formulation. Therefore, FTIR analysis alone cannot conclusively distinguish covalent conjugation from electrostatic interactions or hydrogen bonding between the carboxyl groups of folic acid and the amino groups of chitosan [19,49].

Since no chemical coupling agents were used during formulation, folic acid is more appropriately described as surface-associated or surface-functionalized rather than covalently conjugated. Although the FTIR spectra support interaction between folic acid and chitosan, additional analytical techniques such as NMR, XPS, or dedicated conjugation studies would be required to conclusively demonstrate covalent bond formation.

#### 2.2.6. Differential Scanning Calorimetry (DSC)

**Figure 6:**
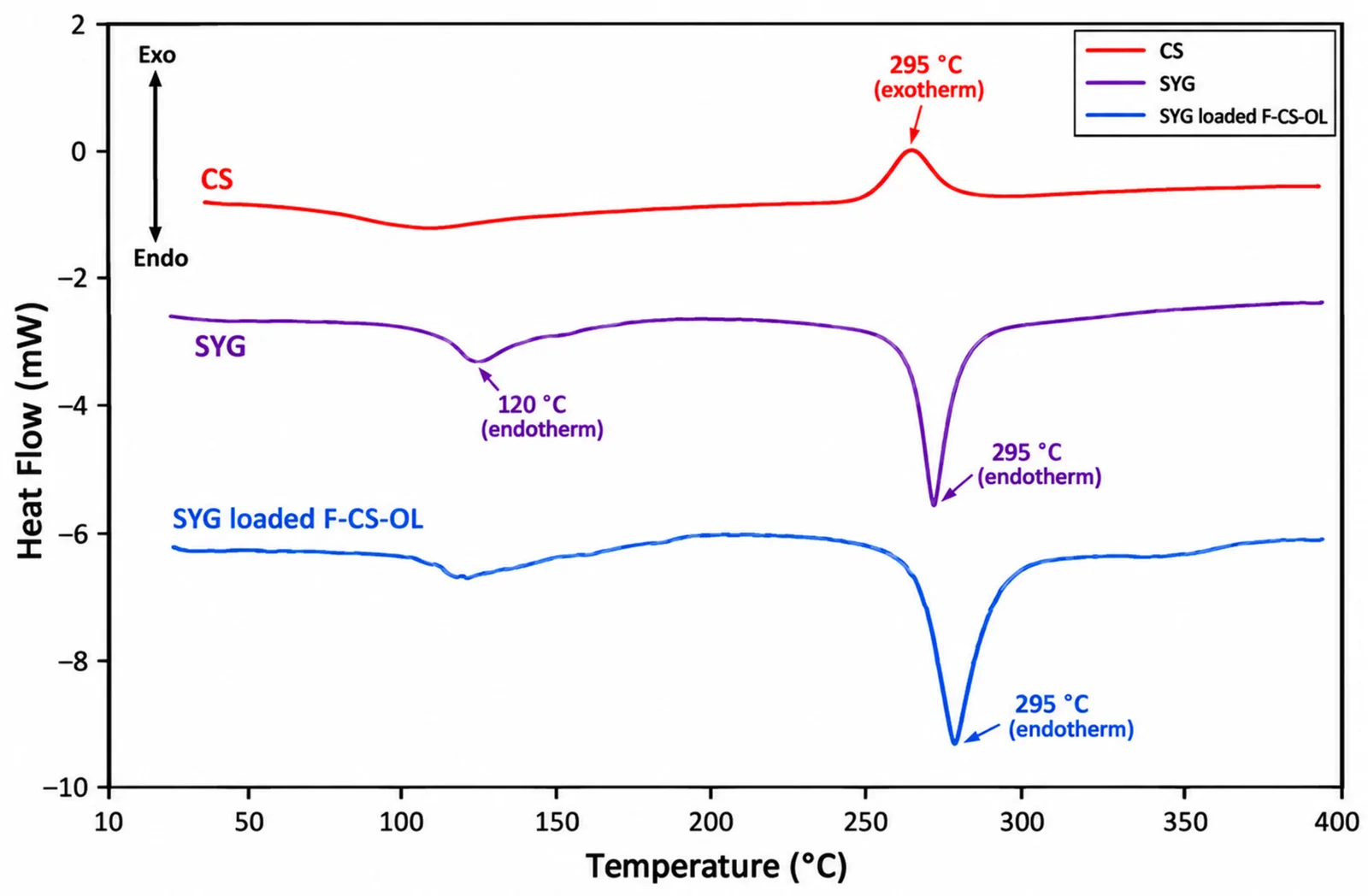
DSC thermograms of SYG, CS, and SYG-loaded F-CS-OL. Heat flow (mW) was recorded as a function of temperature over the range of 10–350 °C. The direction of endothermic and exothermic transitions is indicated in the figure.

DSC is a widely used thermal analysis technique for evaluating the thermal behavior of materials and provides valuable information regarding phase transitions, crystallinity, thermal stability, and component interactions within pharmaceutical systems [38]. As shown in Figure 6, CS exhibited a broad endothermic event at approximately 100 °C, which can be attributed to the loss of bound water, followed by a characteristic exothermic peak near 295 °C associated with polymer degradation [50,51]. The thermogram of SYG showed a broad endothermic transition centered around 120 °C, reflecting the complex multicomponent nature of the extract rather than a distinct melting event. In contrast, the thermogram of the SYG-loaded F-CS-OL formulation displayed thermal behavior that differed from those of the individual components. A broad endothermic transition was observed in the high-temperature region where pure chitosan exhibited its degradation exotherm. This observation suggests that incorporation of SYG into the oleosomal system and coating with chitosan and folic acid modified the thermal properties of the individual components. Since the final formulation is a multicomponent system, the observed thermal events likely represent overlapping transitions arising from interactions among the phospholipid matrix, oleic acid, CS coating, folic acid, and encapsulated SYG rather than the behavior of any single component. The alteration, broadening, and shifting of the characteristic thermal events of the individual materials support successful incorporation of SYG within the carrier system and indicate improved compatibility among the formulation components, rather than the presence of a simple physical mixture.

#### 2.2.7. Stability

The physical stability of F6 and F7 during storage at 4 ± 1 °C was investigated by monitoring changes in particle size, PDI, zeta potential, and EE% over a period of 3 months (Table 3). Only slight increases in particle size were observed during storage. The mean diameter of F7 increased from 80.78 ± 1.02 nm in the freshly prepared formulation to 81.56 ± 0.87 nm after 1 month and 83.41 ± 0.58 nm after 3 months. Similarly, F6 showed a minor increase in particle size from 61.60 ± 0.34 nm to 63.67 ± 1.03 nm after 3 months. The limited increase in vesicle size suggests the absence of significant aggregation or fusion phenomena during refrigerated storage.

The PDI values remained below 0.3 throughout the study period for both formulations, indicating maintenance of a relatively uniform particle size distribution. For F7, the PDI increased only slightly from 0.227 ± 0.012 to 0.232 ± 0.03 after 3 months, while F6 exhibited a similarly small increase from 0.263 ± 0.013 to 0.279 ± 0.01.

Likewise, zeta potential values showed negligible changes during storage. F7 maintained a negative surface charge ranging from −15.5 ± 1.12 mV initially to −15.1 ± 1.56 mV after 3 months, while F6 remained between −14.7 ± 1.89 mV and −14.3 ± 1.28 mV. The stability of the zeta potential values indicates preservation of the folic acid/chitosan coating layer and suggests that no appreciable changes in surface characteristics occurred during storage.

Assessment of drug retention demonstrated preliminary formulation stability. The EE% of F7 decreased marginally from 94.98 ± 0.56% in the freshly prepared formulation to 93.87 ± 0.34% and 93.34 ± 0.76% after 1 and 3 months, respectively. The small reduction in EE% (<2%) indicates minimal drug leakage during storage and may be attributed to the stabilizing effect of the chitosan coating, which has been reported to reduce membrane permeability and retard drug diffusion from lipid-based nanocarriers [43].

Overall, the findings demonstrate that the F-CS-OL retained their colloidal characteristics and drug loading capacity throughout the storage period, confirming good physical stability under refrigerated conditions and supporting their suitability for further pharmaceutical development.

### 2.3. In Vivo

The adjuvant-induced arthritis model is well known for its ability to produce persistent inflammatory responses in different rat strains. Experimental arthritis was induced using CFA is composed of inactivated and dried Mycobacterium tuberculosis in mineral oil [43]. The present study utilized a CFA-induced knee arthritis model in which treatment was initiated on Day 4 after arthritis establishment and continued until Day 14. Accordingly, the results reflect the therapeutic effects of the tested formulations during the early phase of experimental arthritis. Although this model reproduces several inflammatory and pathological features relevant to rheumatoid arthritis, it does not fully mimic the chronic, heterogeneous, and progressive nature of established human rheumatoid arthritis. Therefore, the findings should be interpreted within the context of the experimental model employed.

The choice of the oral route for administration of the F-CS-OL is due to ease of dose administration, patient compliance and the oral route offers great flexibility in the dosage regimen [52]. The reported dose of 200 mg/kg refers to the amount of SYG seed extract administered. For the SYG-loaded F-CS-OL group, the amount of formulation administered was calculated according to the experimentally determined drug loading of F7 (16.19%) to provide an extract-equivalent dose of 200 mg SYG/kg body weight. The blank carrier group received the corresponding unloaded formulation.

#### 2.3.1. Assessment of Joint Diameter, Morphology and Arthritis Index

**Figure 7:**
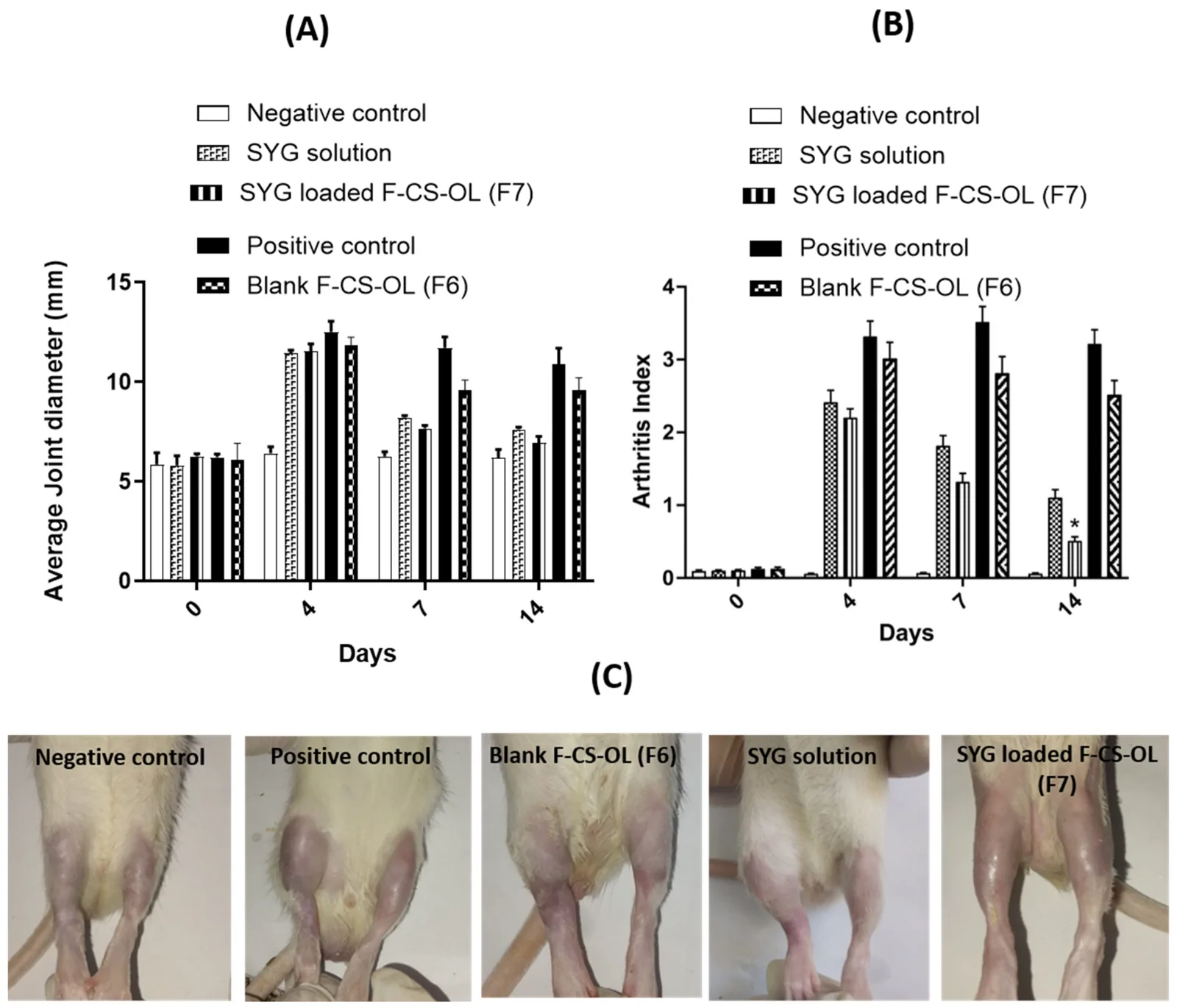
Effect of the SYG-loaded F-CS-OL on arthritis severity. (**A**) Changes in average joint diameter (mm) over 14 days in the negative control, positive control, SYG solution, blank F-CS-OL (F6), and SYG-loaded F-CS-OL (F7) groups (*n* = 5). (**B**) Macroscopic evaluation of Arthritis Index across experimental groups over a 14-day observation period. Clinical arthritis severity was scored on a scale of 0 to 4 based on joint swelling, erythema, and rigidity on Days 0, 4, 7, and 14 of the experiment. Data are represented as mean ± SEM (*n* = 5). (**C**) Representative photographs showing joint shape in the corresponding groups. Quantitative swelling measurements were performed at the right knee joint, which was the site of CFA administration. The photographs are presented only as representative visual documentation of the hind limb appearance and are not intended for quantitative assessment.

Figure 7A describes the average joint diameter (mm) measured over 14 days, with time points at day 0, 4, 7, and 14. Baseline measurements were obtained on day 0 before CFA administration. Treatment was initiated on day 4 after confirmation of arthritis development; therefore, day 4 measurements represent pre-treatment values. No treatment was administered prior to day 4, and no intermediate measurements were collected between days 4 and 7. The negative control remains near baseline throughout, indicating minimal or no inflammatory swelling, while the positive control shows a marked increase in joint diameter, peaking around day 4 and remaining elevated afterward. The SYG solution reduces swelling to some extent, but SYG-loaded F-CS-OL (F7) produced a stronger and more sustained reduction in joint enlargement than the solution alone. The blank F-CS-OL (F6) also shows a partial effect, suggesting that the carrier itself may contribute some anti-inflammatory benefit.

Macroscopic scoring of joint disease progression over the 14-day study period demonstrated robust disease induction and clear differences in therapeutic response among treatment formulations (Figure 7B). On Day 0, baseline clinical scores remained near zero across all experimental groups, confirming the absence of pre-existing inflammation. By day 4 post-induction, a marked elevation in clinical arthritis scores was evident in all arthritic groups, confirming successful disease induction. The untreated Positive Control group peaked at day 4 and continued throughout the experiment, reflecting persistent, severe joint inflammation. Similarly, the Blank F-CS-OL (F6) vehicle control exhibited minimal therapeutic effect, maintaining high arthritis scores throughout the study period. Treatment with both SYG solution and SYG-loaded F-CS-OL (F7) initiated a time-dependent alleviation of joint inflammation starting from Day 4 onward. Clinical scores in the F7 group decreased sharply by Day 14.

Figure 7C demonstrates the joint morphology at the end of the experiment from each group and supports the quantitative data on joint diameter in Figure 7A. The negative control shows the least obvious swelling, whereas the positive control displays pronounced paw and ankle enlargement, consistent with severe arthritic inflammation. The blank F-CS-OL group shows intermediate improvement, and the SYG solution shows visible attenuation of swelling compared with the positive control. The SYG-loaded F-CS-OL group showed the best visual reduction in edema and joint deformity, indicating improved therapeutic performance of the SYG when encapsulated in F-CS-OL.

#### 2.3.2. Biochemical Analysis

The current study assessed the therapeutic efficacy of a nano-encapsulated SYG extract formulation in an experimental RA model, with a focus on a broad range of mediators including inflammation, angiogenesis, adhesion, matrix degradation, and B-cell chemoattraction. RA is categorized as a chronic inflammatory illness and an autoimmune disease. RA aetiology includes raised levels of surface adhesion molecules and chemokines in addition to increased local fibroblast activation. These factors, when paired with local hypoxia, cause angiogenesis and lymphangitic dysfunction, establishing the early hallmarks of synovitis. However, the exact cause of RA remains unknown [1]. Furthermore, RA progression is linked to elevated levels of chemokines and proinflammatory cytokines [53]. Our findings support this paradigm, as the positive control (CFA-induced arthritis) group showed significant elevations in all measured parameters (TNF-α, IL-6, CRP, VEGF, ICAM-1, MMP-3, and CXCL13) compared to the negative control. These findings further confirm the establishment of a robust inflammatory and tissue-destructive response in the CFA-induced arthritis model.

Although VEGF, ICAM-1, MMP-3, and CXCL13 are well-recognized biomarkers associated with angiogenesis, leukocyte recruitment, matrix degradation, and lymphoid organization, respectively, serum measurements alone do not establish direct inhibition of these tissue-level processes. Therefore, the present findings should be interpreted as demonstrating modulation of biomarkers associated with these pathways rather than definitive mechanistic inhibition.

##### Suppression of the TNF-α/IL-6/CRP Inflammatory Axis by SYG-Loaded F-CS-OL

**Figure 8:**
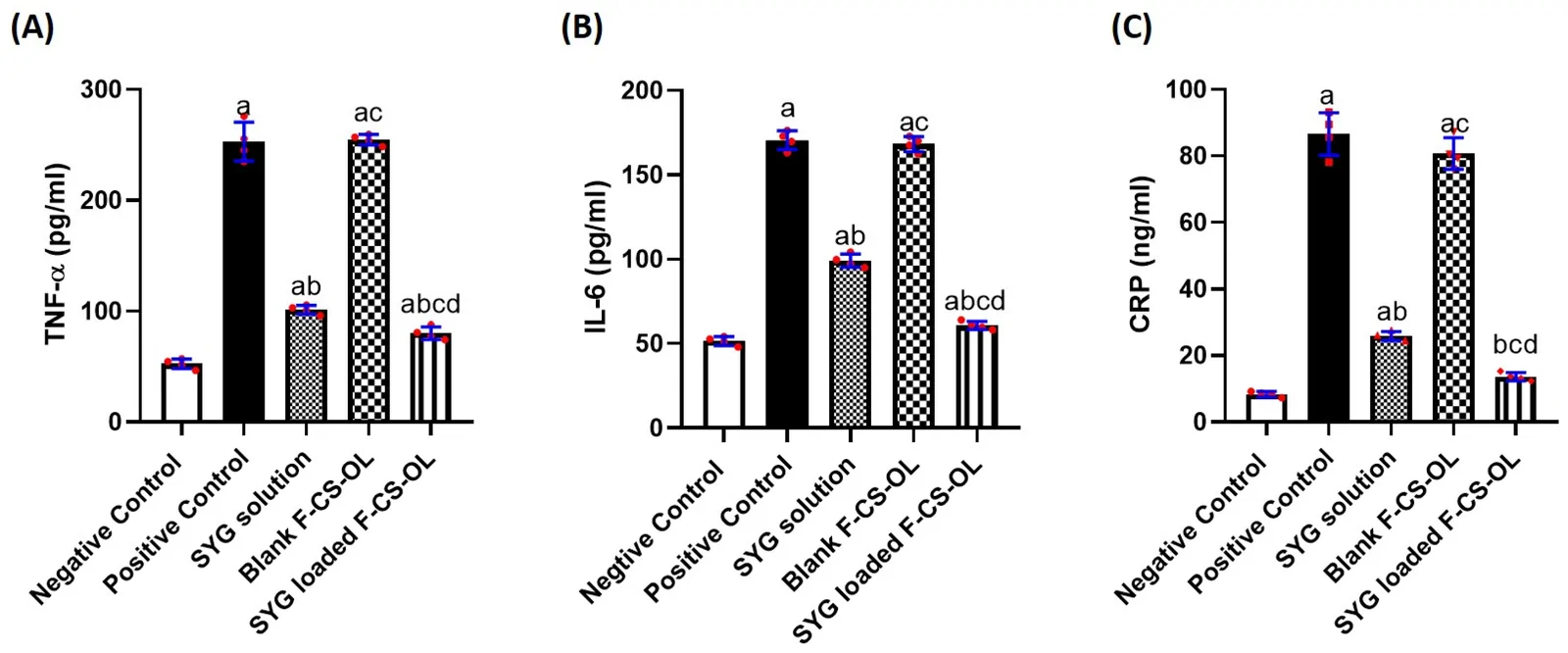
Effect of SYG-loaded F-CS-OL therapy on the TNF-α/IL-6/CRP inflammatory axis. Serum levels of (**A**) tumor necrosis factor-alpha (TNF-α), (**B**) interleukin-6 (IL-6), and (**C**) C-reactive protein (CRP). Data were presented as the mean ± SEM (*n* = 4/group contributing analyzable serum samples) and were analyzed using one-way ANOVA followed by Tukey’s multiple comparison test, *p* < 0.05. The letters (a, b, c and d) represent significant differences from the negative control group, positive control group, SYG solution group and blank F-CS-OLs group, respectively.

The systemic anti-inflammatory efficacy of the SYG-loaded F-CS-OL formulation was assessed by measuring serum levels of TNF-α, IL-6, and CRP in all experimental groups (Figure 8). All three inflammatory mediators were significantly higher in the positive control group than in the negative control group. In particular, levels of TNF-α, IL-6, and CRP were elevated by roughly 4.7, 3.3, and 9.8 times, respectively (*p* < 0.0001), indicating the development of a robust systemic inflammatory state. The marked increase in CRP observed in the CFA-induced arthritis group reflects the strong systemic acute-phase response associated with adjuvant-induced inflammation. Although treatment with SYG-loaded F-CS-OL significantly reduced CRP levels, these findings should be interpreted within the context of the experimental model. Because CRP elevations in CFA-induced arthritis may be more pronounced than those typically observed in human rheumatoid arthritis, the magnitude of CRP reduction reported herein should not be directly extrapolated to clinical disease control.

The free extract group demonstrated a moderate but significant reduction in all three parameters in comparison with the positive control group. The nano-loaded extract group significantly reduced TNF-α, IL-6, and CRP levels by approximately 68%, 64%, and 84% compared to the positive control group, respectively. Nevertheless, compared to the free extract group, the nano-loaded extract group significantly reduced TNF-α, IL-6, and CRP serum levels (*p* = 0.0238, <0.0001, and 0.0022, respectively).

Nano-encapsulation significantly improved the ability of the SYG extract to suppress the TNF-α/IL-6/CRP inflammatory axis. Notably, CRP levels in the nano-loaded group approached those of the negative control (y9).

The SYG-loaded F-CS-OL formulation outperformed the free extract in all measured outcomes. This increased potency is consistent with the known advantages of nano-delivery platforms, including enhanced extract solubility, prolonged circulation time, targeted tissue accumulation, and sustained release kinetics, which collectively maximize pharmacological action at the site of inflammation [7,54].

Herein, the SYG-loaded F-CS-OL effectively suppressed the TNF-α/IL-6/CRP inflammatory axis, resulting in near-normalization of CRP and IL-6 levels and significant reduction in TNF-α. The interaction of these three molecules is central to RA pathogenesis. CRP is a highly conserved acute-phase inflammatory protein produced primarily by the liver in response to increased levels of circulating inflammatory cytokines [55].

CRP is essential for cellular activation and cytokine synthesis. Two important cytokines in the pathophysiology of RA are TNF-α and IL-6. In a thorough analysis of CRP and cytokines in RA, Alunno et al. discovered that because IL-6 and CRP co-regulate and are frequently elevated in reaction to one another, it is challenging to distinguish their functions in RA; the same is true for TNF-α. Therefore, it is challenging to differentiate between these three molecules [56].

Our results support this interdependence, as the elevation and subsequent coordinated suppression of all three mediators followed parallel trajectories. Furthermore, CRP reduction is associated with reduced disease activity [57], so the reduction in CRP levels suggests attenuation of the systemic inflammatory response in the CFA-induced arthritis model, although direct effects on hepatic acute-phase responses were not investigated.

##### Reduction in VEGF and ICAM-1 Levels Associated with Angiogenic and Inflammatory Pathways by SYG-Loaded F-CS-OL

Angiogenesis was assessed indirectly through serum VEGF and ICAM-1 measurements. Although VEGF is a recognized biomarker associated with angiogenic activity, direct assessment of synovial neovascularization was not performed. Therefore, the present findings should be interpreted as demonstrating modulation of angiogenesis-associated biomarkers rather than direct evidence of reduced microvessel formation. Future studies employing CD31 immunohistochemistry, microvessel density analysis, and tissue-based assessment of VEGF, ICAM-1, MMP-3, and CXCL13 are warranted.

**Figure 9:**
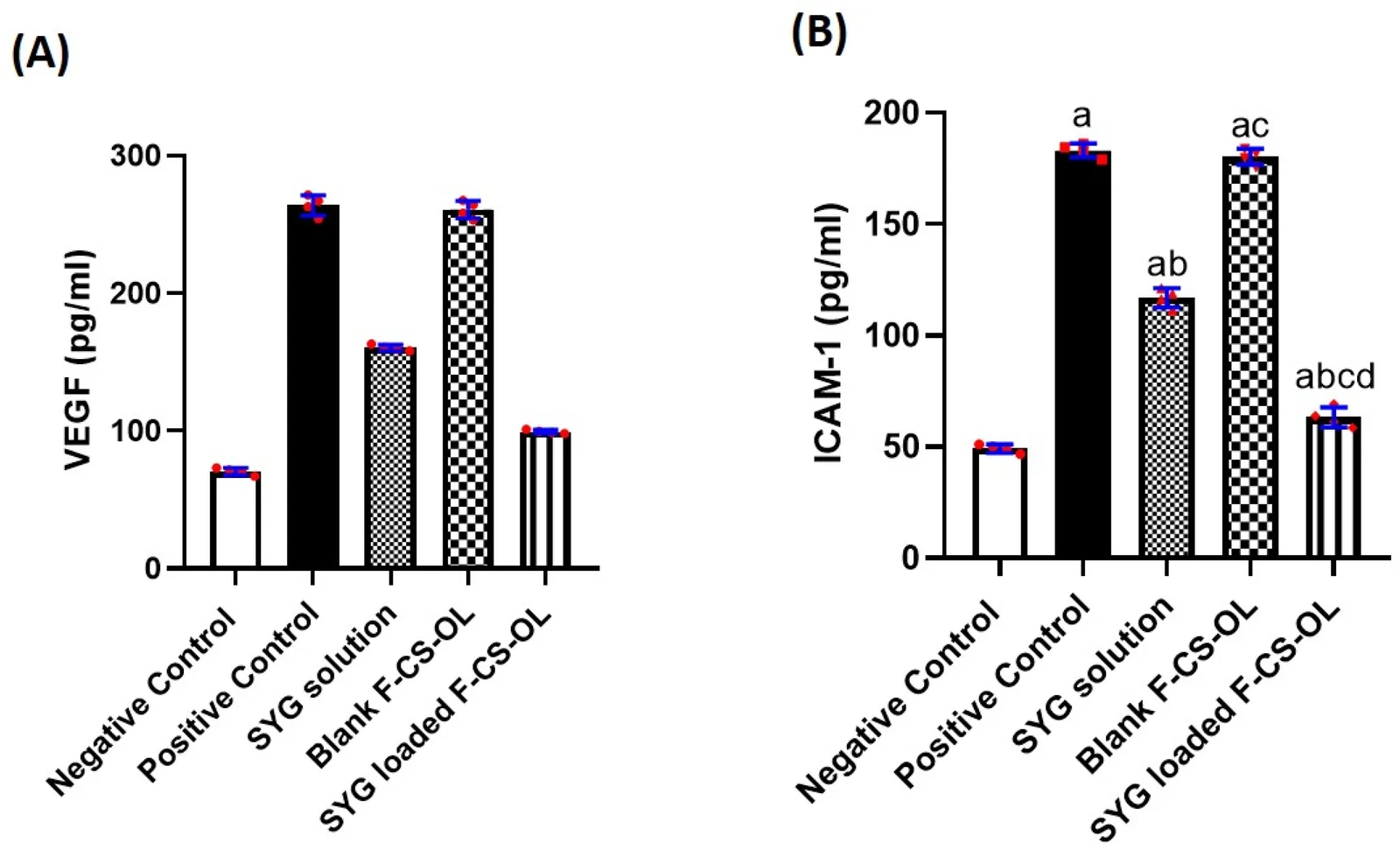
Effect of SYG-loaded F-CS-OL therapy on VEGF and ICAM-1 levels associated with angiogenic and inflammatory pathways. Serum concentrations of (**A**) vascular endothelial growth factor (VEGF) and (**B**) intercellular adhesion molecule-1 (ICAM-1). Data were presented as the mean ± SEM (*n* = 4/group contributing analyzable serum samples) and were analyzed using one-way ANOVA followed by Tukey’s multiple comparison test, *p* < 0.05. The letters (a, b, c and d) represent significant differences from the negative control group, positive control group, SYG solution group and blank F-CS-OLs group, respectively.

The positive control group had significantly higher levels of VEGF (3.7-fold) and ICAM-1 (3.7-fold) (*p* < 0.0001) compared to the negative control group, indicating increased angiogenic signaling and endothelial activation associated with RA synovial pathology (Figure 9).

When compared to the positive control group, the free extract treatment significantly attenuated these mediators, lowering VEGF and ICAM-1 levels (*p* < 0.0001). The nano-loaded extract formulation produced the largest reduction in both VEGF and ICAM-1 levels, corresponding to reductions of roughly 62% and 65% from the positive control, respectively (*p* < 0.0001), in line with the pattern seen for the inflammatory cytokines. These results demonstrated significant reductions in circulating VEGF and ICAM-1 levels, biomarkers associated with angiogenic signaling and leukocyte–endothelial interactions. In comparison to the free extract group, the SYG-loaded F-CS-OL group considerably reduced the serum levels of VEGF and ICAM-1 (*p* < 0.0001) (Figure 9).

The SYG-loaded F-CS-OL formulation also showed a potent dual inhibitory effect on VEGF and ICAM-1, which are important mediators of pathological angiogenesis and immune cell function, respectively. Angiogenesis is required for the expansion of synovial tissue in RA, allowing leukocyte recruitment, synovial hyperplasia, and the formation of aggressive pannus tissue [1,5]. Our findings revealed that VEGF levels were significantly elevated in the RA group and significantly reduced by the nano-loaded treatment. Interestingly, a number of associations between CRP and VEGF have been found in RA.

In a recent meta-analysis of 165 studies, Zhan et al. found that VEGF correlated weakly with disease activity (DAS28) and CRP (r = 0.36) [58]. Studies that demonstrate that adding CRP to HUVEC cells (an endothelial cell line) causes a 10-fold increase in the adhesion molecule ICAM-1 and a several-fold increase in VCAM-1 support the idea that CRP may contribute to the inflammatory aspect of atherosclerosis by promoting cell adhesion to the endothelial space [55,58].

Our findings apply this observation to the RA context, where the simultaneous reduction in CRP, VEGF, and ICAM-1 by the nano-loaded extract suggests a coordinated de-escalation of the CRP-VEGF-ICAM-1 signaling axis. Activated macrophages primarily target RA sites through inflammation-induced binding before extravasating and adhering to the inflamed pannus of blood vessels via highly expressed adhesion molecules such as ICAMs [5]. The simultaneous reduction in VEGF and ICAM-1 levels suggests modulation of pathways associated with angiogenesis and leukocyte recruitment in CFA-induced arthritis. However, direct inhibition of these tissue-level processes was not evaluated in the present study.

##### Reduction in MMP-3 and CXCL13 Levels Associated with Joint Destruction and Adaptive Immune Responses by SYG-Loaded F-CS-OL

**Figure 10:**
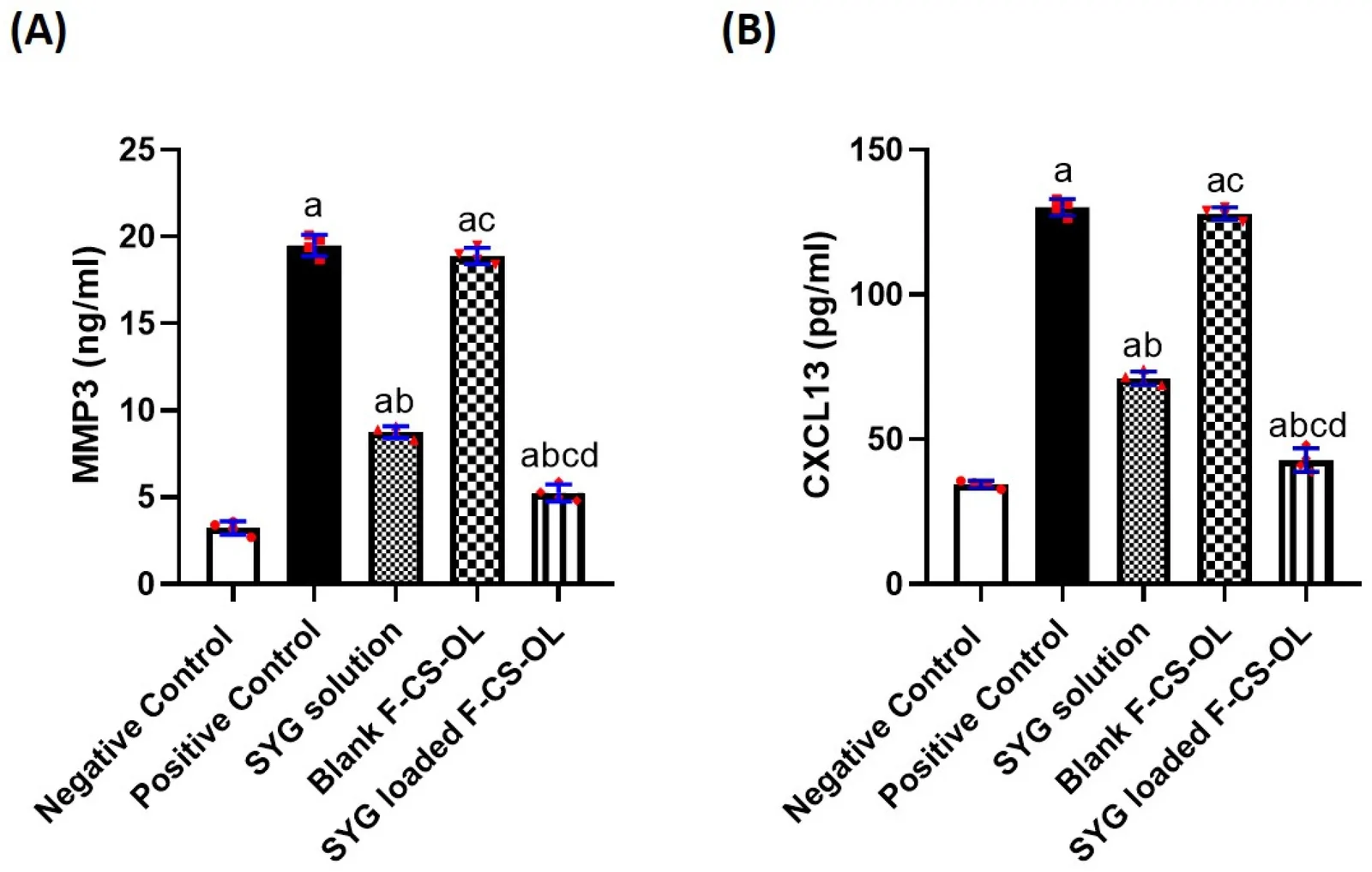
Effect of SYG-loaded F-CS-OL therapy on MMP-3 and CXCL13 levels associated with joint destruction and adaptive immune responses. Serum levels of (**A**) matrix metalloproteinase-3 (MMP-3) and (**B**) C-X-C motif chemokine ligand 13 (CXCL13). Data were presented as the mean ± SEM (*n* = 4/group contributing analyzable serum samples) and were analyzed using one-way ANOVA followed by Tukey’s multiple comparison test, *p* < 0.05. The letters (a, b, c and d) represent significant differences from the negative control group, positive control group, SYG solution group and blank F-CS-OLs group, respectively.

To assess the impact on downstream joint destruction and adaptive immune organization, serum levels of MMP-3, a key effector of cartilage degradation, and CXCL13, a B-cell chemoattractant critical for ectopic lymphoid structure formation, were measured (Figure 10). The positive control group showed a significant 6-fold increase in MMP-3 (*p* < 0.0001) and a 3.8-fold increase in CXCL13 (*p* < 0.0001) compared to the negative control, indicating ongoing matrix degradation and increased B-cell recruitment signals.

When compared to the positive control group, the free extract group significantly reduced MMP-3 and CXCL13. Critically, the SYG-loaded F-CS-OL formulation resulted in the greatest reduction in both parameters. MMP-3 and CXCL13 were reduced by 73% and 67%, respectively, in comparison with the positive control and free extract group (*p* < 0.0001). The reduction in serum levels of MMP-3 and CXCL13 by SYG-loaded F-CS-OL therapy suggests a potential impact on these pathways, which orchestrate ectopic lymphoid neogenesis in the inflamed synovium.

MMP-3 and CXCL13 are downstream effectors of joint destruction and adaptive immune organization, respectively. MMP-3 is a key matrix metalloproteinase involved in the degradation of cartilage proteoglycans and ECM components in RA, and high levels are strongly linked to radiographic progression [2,6]. Our outcomes showed that MMP-3 increased approximately 6-fold in the RA group, but was reduced by 73% in the nano-loaded group, outperforming the free extract. This finding suggests a potential reduction in biochemical markers associated with joint tissue degradation.

CXCL13 levels were found to be higher in RA compared to normal conditions, facilitating TNF-α synthesis in synovial fibroblasts through interaction with the CXCR5 receptor [59]. Unlike other chemokine receptors, CXCL13 only acts as a chemotactic agent for CXCR5, indicating a highly specific signaling axis [9]. Results of the present study confirmed a significant increase in CXCL13 in the RA group, which is substantially suppressed by the SYG-loaded F-CS-OL. Evidence suggests that CXCL13 activates intracellular signaling pathways, such as NF-κB. NF-κB is a transcription factor that controls pro-inflammatory genes like IL-6, TNF-α, and IL-1β, regulating inflammation development [60]. The CXCL13-CXCR5 axis is critical for the formation of ectopic lymphoid structures within the inflamed synovium, which serve as sites for B-cell activation and autoantibody production, exacerbating the autoimmune pathology [8,9]. Thus, the observed reduction in CXCL13 levels may reflect modulation of signaling pathways associated with B-cell recruitment and lymphoid organization. The coordinated reduction in MMP-3 and CXCL13 suggests modulation of biomarkers associated with joint tissue remodeling and adaptive immune responses in CFA-induced arthritis.

Given the reported involvement of the CXCL13-CXCR5 axis in inflammatory signaling and lymphoid organization, the observed reduction in CXCL13 levels may be associated with modulation of these pathways. However, because CXCR5 expression and NF-κB activity were not assessed in the present study, any involvement of the CXCL13-CXCR5/NF-κB pathway should be regarded as a proposed mechanism based on previous literature rather than a directly demonstrated effect of SYG-loaded F-CS-OL.

Finally, serum biomarker changes indicate systemic alterations but do not directly affect tissue-level processes like angiogenesis, leukocyte infiltration, cartilage degradation, or lymphoid neogenesis. Future research should incorporate direct histological, immunohistochemical, or functional assays to assess these outcomes at the tissue level and to support the proposed mechanisms.

#### 2.3.3. Histopathological Examination

**Figure 11:**
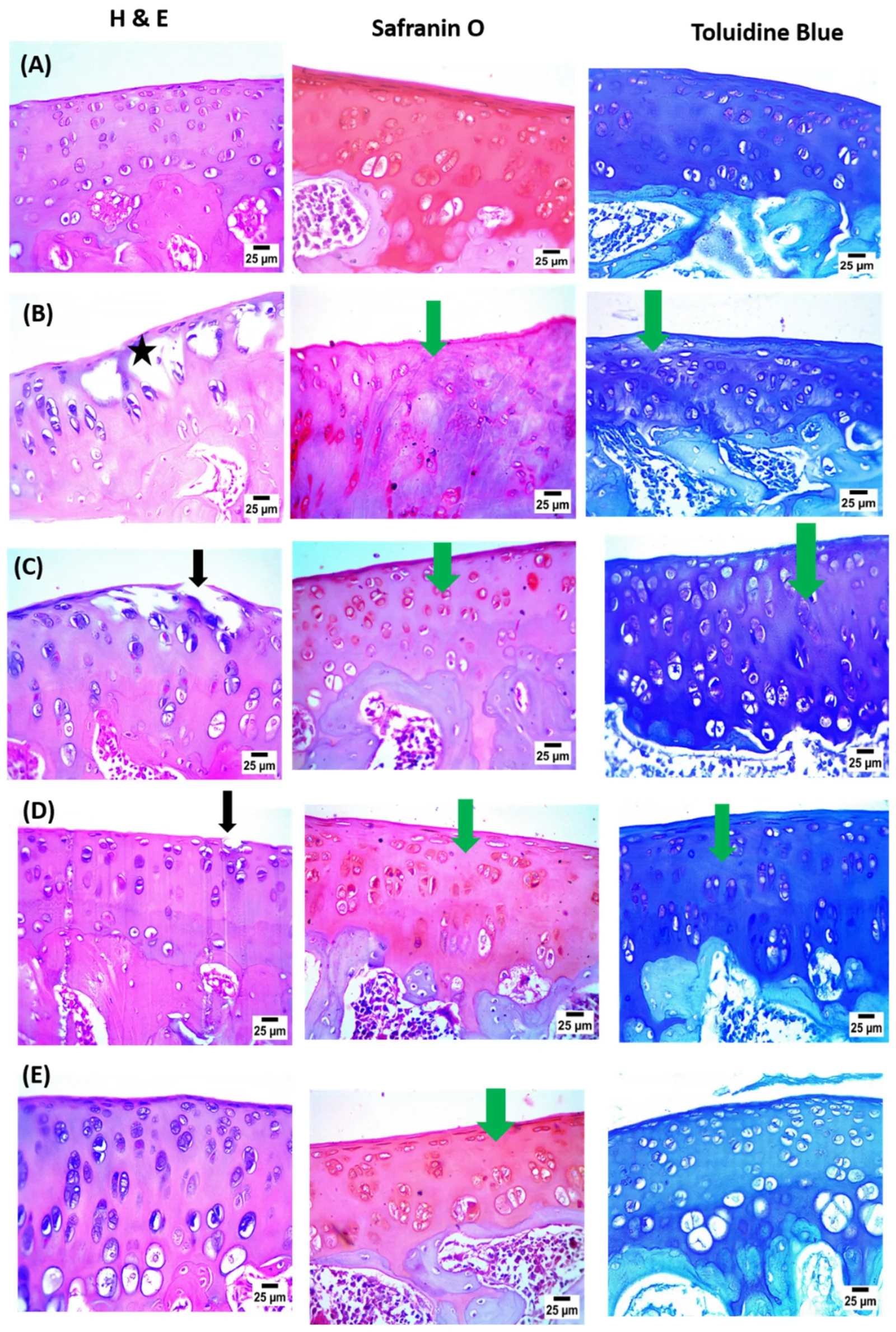
Histopathological examination of dissected joints at the end of the experiment for different experimental groups after staining with H&E stain, Safranin O and Toluidine blue stain. (**A**) negative control, (**B**) positive control, (**C**) blank F-CS-OL, (**D**) SYG solution and (**E**) SYG-loaded F-CS-OL. Black arrows in H&E sections demonstrate loss of the superficial layer. Stars demonstrate loss of the superficial and transitional layers of the articular surface. Green arrows in Toluidine blue and Safranin O staining represent the uneven staining and reduction in proteoglycans. Scale bars are 25 µm with magnification 400×.

As demonstrated in Figure 11. In H and E staining, the negative control group demonstrated normal joint structure with no marked pathological changes in the articular surface. On the other hand, the positive control group illustrated severe loss of superficial and transitional layers of the articular surface (star) and destruction of the superficial layer in the articular surface. Moreover, the groups treated with blank F-CS-OL and SYG solution showed moderate and mild loss of articular surface, respectively. Meanwhile, the group treated with SYG-loaded F-CS-OL demonstrated near-normal histological features in the articular surface.

In both Safranin O and toluidine blue stains, the negative control group showed normal structure with even stain distribution, and this was also noted in the group treated with SYG-loaded F-CS-OL. On the contrary, the positive control demonstrated severe reduction in proteoglycan content in the articular surface (uneven staining). Meanwhile, the groups treated with Blank F-CS-OL and SYG extract demonstrated moderate to mild loss of proteoglycan, respectively.

Histopathological examination results are presented along with ELISA and joint diameter measurements. All results demonstrated the superiority of SYG-loaded F-CS-OL over SYG solution and its corresponding blank formulation. This may be ascribed to the improvement of SYG pharmacological properties upon its nanoencapsulation.

The blank F-CS-OL formulation showed limited effects that were observable in some physical and histopathological parameters but did not result in significant changes in the measured inflammatory biomarkers, as histological improvements were more evident than biochemical improvements in the blank carrier group.

Although oleic acid [15] and CS [43] have been reported to exhibit anti-inflammatory and antioxidant activities in previous studies, the blank F-CS-OL formulation did not significantly alter the measured serum biomarkers in the present study. Nevertheless, modest improvements in joint swelling and histopathological findings were observed; this may be ascribed to the dose, formulation composition or the duration of treatment. Therefore, the carrier may contribute to certain aspects of the observed response, but the pronounced therapeutic effects achieved with SYG-loaded F-CS-OL cannot be explained by carrier-associated effects alone.

Therefore, the enhanced efficacy of SYG-loaded F-CS-OL is likely attributable to a combination of carrier-associated biological effects and the pharmacological activity of the encapsulated SYG extract. Nevertheless, the substantially greater improvement achieved with the loaded formulation compared with the blank carrier suggests an additional therapeutic contribution from SYG and/or its enhanced delivery through the targeted nanocarrier system. This coincides with the previous reports on the ability of nanoencapsulation to enhance the efficacy and pharmacological effects of the loaded cargos from natural origin in different applications [16,61] including RA [62].

Histopathological findings demonstrated variable degrees of protection among the treatment groups. Both the blank F-CS-OL and SYG solution groups exhibited partial improvement compared with the positive control group. Interestingly, the blank carrier demonstrated better preservation of tidemark integrity than the free extract, although cellular abnormalities remained evident. The SYG-loaded F-CS-OL group showed the most favorable overall histological profile (Table 4); however, the histological findings should be interpreted in conjunction with the corresponding Mankin scores rather than qualitative descriptors alone.

## 3. Materials and Methods

Phospholipon 90H was obtained from Lipoid (Ludwigshafen, Germany). Chitosan (96% degree of deacetylation) and cholesterol were bought from Sigma-Aldrich, Sain Loius, MO, USA. Complete Freund’s adjuvant (Catalog number F5881) was purchased from Sigma-Aldrich, Sain Loius, MO, USA. Folic acid was supplied by Pharco Pharmaceutical Companies (Alexandria, Egypt). All other chemicals were analytical grade. Oleic acid was bought from Nice Chemicals (Kerala, India). All other chemicals were analytical grade.

### 3.1. Preparation of SYG Seed Extract

SYG Skeels seeds were authenticated at the Faculty of Science, Alexandria University, Egypt. A voucher specimen was deposited under voucher number ALEX 4136. The extraction procedure was performed using batches of SYG seed powder prepared according to the protocol described below. A single extract batch was subsequently used for phytochemical analysis, formulation development, and biological evaluation. The preparation of the aqueous extract from SYG seeds was carried out using a standardized procedure to ensure extract quality and reproducibility. Initially, approximately two kilograms of ripe SYG fruits were collected and carefully inspected to remove any damaged or spoiled samples.

The fruit pulp and seeds were carefully separated. To get rid of any pulp residue that might have stuck to the collected seeds, they were once again cleaned with distilled water. After air drying, the seeds were further dried for 24 h at 40 °C in a tray dryer until they reached a consistent weight. Before being extracted, the dried seeds were ground into a fine powder using a lab grinder and kept in sealed containers at 4 °C.

Ten grams of the powdered seed material were combined with distilled water at a sample-to-solvent ratio of 1:10 (*w*/*v*) for the extraction procedure. For 72 h at room temperature, the mixture was continuously shaken at 150 rpm using a mechanical shaker. The mixture was also exposed to ultrasonic treatment for 20 min per day in an ultrasonic bath to boost the release of intracellular phytochemicals and enable the destruction of cellular structures in order to increase the extraction efficiency of bioactive compounds.

Following extraction, solid residues were eliminated by vacuum-filtering the mixture with Whatman No. 1 filter paper. A rotary evaporator operating at 45–50 °C was then used to concentrate the filtrate under lower pressure. After that, the concentrated extract was freeze-dried to produce a dry powder. Before being used in additional experiments, the final extract was kept at 4 °C in airtight amber glass vials. The main phytochemicals found in SYG seeds, such as polyphenols, flavonoids, and tannins, which are in charge of the plant’s biological activity, are effectively extracted and preserved using this technique [63].

The amount of seed powder described in the extraction procedure represents the extraction protocol and not the total quantity of plant material processed for the study. A sufficient quantity of extract was prepared in a single batch and subsequently used for all phytochemical, formulation, characterization, and biological evaluations.

### 3.2. Chemical Characterization of SYG Extract

#### 3.2.1. Vanillin-Reactive Saponin-Equivalent Content Quantification

The modified vanillin–sulphuric acid technique for total vanillin-reactive saponin content (TSC) was used to calculate the total saponin content of SYG seed extract. The calibration curve was built using escin, a naturally occurring combination of triterpenoid saponins, as the reference standard. 150 mg of escin were dissolved in 10 mL of methanol to create a stock solution of escin (15 mg/mL). Methanol was then used to create serial dilutions and as a solvent blank for the standard curve.

In order to assist pre-leaching, 1 g of powdered SYG seed extract and 30 mL of methanol were combined in a 100 mL conical flask and left for 30 min at room temperature. The suspension was then filtered through a 0.45 µm membrane filter. To determine the total saponin content, the resultant clear filtrate was gathered.

Test tubes containing aliquots of the extract and standards were heated to 65 °C in a water bath until all of the methanol had evaporated, which took around five minutes. Each tube was then filled with 0.5 mL of a 4% vanillin solution in methanol (*w*/*v*) and 2.5 mL of 72% sulfuric acid (*v*/*v*). After covering and vortex-mixing the tubes, they were incubated for fifteen minutes at 60 °C in a water bath. The tubes were cooled at room temperature for five minutes following incubation. A Biosystems 310 spectrophotometer was used to measure the absorbance at 560 nm following blank correction. The calibration curve for the measurement of total saponins was created by plotting the absorbance values against the relevant concentrations of the standard solutions.

$$\text{Total vanillin-reactive saponin content}\;=\frac{\text{weight of saponins in the extract}\;(\mathrm{mg})}{\text{weight of powder}\;(g)}\times (1-(\mathrm{moisture}\%/100))$$

The TSC was represented as mg escin equivalents per gram dry weight of powder SYG seed extract (mg AE/gm).

#### 3.2.2. Total Tannin Quantification

After a spectrophotometric process, the Folin-Denis method was used to quantify the total tannin content of SYG seed extract against a tannic acid calibration curve. Results were expressed as tannic acid equivalents (TAE). To create a calibration curve, standard tannin solutions were made at various concentrations. A suitable quantity of powdered SYG was extracted with ethanol and filtered to produce a clear solution in order to prepare the extract. Following the transfer of an aliquot of the extract into test tubes, the Folin-Denis reagent and sodium carbonate solution were added to start the reaction. For adequate color development, the mixture was properly combined and left to stand at room temperature.

The strength of the blue complex that results from tannins reacting with the Folin-Denis reagent is directly correlated with the concentration of tannins. A Biosystems 310 spectrophotometer was used to evaluate the absorbance of the resultant solution at 760 nm in comparison to a reagent blank. Plotting absorbance measurements against the respective tannin standard concentrations produced a calibration curve. Next, using the standard curve’s regression equation, the extract’s total tannin content was determined and represented as milligrams of tannin equivalents per gm dry extract [64].

#### 3.2.3. Determination of Total Phenolic Content (TPC)

Total phenolic content was determined using the Folin–Ciocalteu colorimetric method according to Singleton and expressed as mg gallic acid equivalents (GAE) per gram of dry extract [65]. In short, two milliliters of methanol were used to dissolve one milliliter of extract. A total of 2.5 mL of ten-fold diluted Folin–Ciocalteu reagent was combined with an aliquot of 500 µL of the produced extract solution. Next, 2.5 mL of sodium carbonate solution (75 g/L) was added. To ensure full color development, the reaction mixture was vortexed for around ten seconds before being incubated at 25 °C for two hours.

Following incubation, a UV-Vis spectrophotometer was used to determine the absorbance at 765 nm in comparison to a reagent blank. A calibration curve made with gallic acid was used to measure the total phenolic content, which was then reported as milligrams of gallic acid equivalents per gram of dry extract (mg GAE/g).

#### 3.2.4. Determination of Total Flavonoid Content (TFC)

A modified aluminum chloride colorimetric technique was used to determine the extracts’ total flavonoid concentration. In a 10 mL volumetric flask, 1 mL of the extract was first dissolved in 2 mL of methanol. Distilled water was used to create reagent solutions of 5% sodium nitrite (NaNO_2_), 7% aluminum chloride (AlCl_3_), and 5% sodium hydroxide (NaOH).

200 µL of the extract solution and 75 µL of a 5% sodium nitrite solution were added to a sealed glass vial for the test. For five minutes, the mixture was left to stand at room temperature. Then, 1.25 mL of aluminum chloride solution and 0.5 mL of sodium hydroxide solution were added. After that, the reaction mixture was sonicated and allowed to generate the flavonoid–aluminum combination for a further five minutes at room temperature.

Following incubation, a UV-Vis spectrophotometer was used to measure the absorbance of the reaction mixture and the standard solutions at 510 nm, using methanol as the blank. Using a standard calibration curve made using quercetin, the total flavonoid concentration was determined. The results were reported as milligrams of quercetin equivalents per gram of dry extract (mg QE/gm dry extract).

#### 3.2.5. ABTS Radical Scavenging Activity

The ABTS radical scavenging activity was evaluated using the method previously reported by Re et al. [66] with slight modifications. The radical solution was prepared using 2,2′-azino-bis (3-ethylbenzothiazoline-6-sulfonic acid), which was dissolved in distilled water to obtain a 7 mM stock solution. The ABTS radical cation (ABTS•^+^) was generated through the reaction between the ABTS solution and Potassium persulfate (2.45 mM). For 12 to 16 h, the reaction mixture was left at room temperature in the dark to allow the radical cation to fully develop. An absorbance of 0.70 ± 0.02 at 734 nm was obtained by diluting the ABTS solution with distilled water before examination at different concentrations (1.95, 3.9, 7.8, 15.62, 31.25, 62.5, 125, 250, 500, and 1000 µg/mL). Three milliliters of the prepared ABTS radical solution and 0.07 milliliters of the plant extract made up the reaction mixture for the experiment. A UV-Vis spectrophotometer was used to measure the absorbance at 734 nm after the mixture was incubated at room temperature for six minutes.

The percentage inhibition of the ABTS radical was calculated using the following equation:

$$\text{Scavenging activity}\;(\%)\;=\;\frac{\mathrm{Acontrol}-\mathrm{Asample}}{\mathrm{Acontrol}}\;\times \;100$$

The dose–response curve was used to calculate the IC_50_ value, which is the extract concentration needed to scavenge 50% of the ABTS radicals. Stronger radical scavenging activity is indicated by lower IC_50_ values, which are frequently employed to assess antioxidant efficacy. Trolox was used as the reference antioxidant for the ABTS assay.

#### 3.2.6. DPPH Radical Scavenging Activity

The 1,1-diphenyl-2-picrylhydrazyl test was used to assess the plant seed extracts’ capacity to scavenge free radicals. In short, an ethanol solution containing 0.1 mM DPPH was made. Three milliliters of plant extract produced in ethanol at different concentrations (1.95, 3.9, 7.8, 15.62, 31.25, 62.5, 125, 250, 500, and 1000 µg/mL) were combined with one milliliter of the DPPH solution for the analysis. Only extracts that were completely soluble in ethanol were used, and the required concentrations were obtained through serial dilution.

After properly vortexing the reaction solutions, the antioxidant compounds and DPPH radicals were allowed to react for 30 min at room temperature. A UV-Vis spectrophotometer was used to measure the absorbance at 517 nm after incubation. The reference standard was ascorbic acid.

The percentage of DPPH radical scavenging activity was used to express the extracts’ antioxidant capacity. A decrease in the reaction mixture’s absorbance suggests that the tested extract has a greater capacity to scavenge free radicals. The following formula was used to determine the % inhibition of DPPH radicals:

$$\text{DPPH scavenging activity}\;(\%)\;=\;\frac{\mathrm{Acontrol}-\mathrm{Asample}}{\mathrm{Acontrol}}\;\times \;100$$

The IC_50_ value, defined as the concentration of extract required to inhibit 50% of the DPPH radicals, was determined from the logarithmic dose–response inhibition curve. Lower IC_50_ values indicate stronger antioxidant activity of the tested extract.

### 3.3. Preparation of SYG Extract Loaded Nanocarriers

#### 3.3.1. Preparation of Blank and SYG-Loaded Liposomes and Oleosomes (OL)

Blank liposomes were generated using the thin film hydration process as previously reported [17]. In summary, 200 mg phospholipon 90 H, 2 mg cholesterol, and 40 mg Tween 80 were mixed with 2 mL absolute ethanol and swirled on a water bath at 50 °C until complete ethanol evaporation and thin film formation. To prepare oleosomes, the same procedure was followed except that oleic acid was included in the lipid phase in a ratio of 1:4 *w*/*w*; films were rehydrated at 60 °C with 10 mL of deionized water. The mixes were ultrasonically processed (Sonica R 2200 EP S3, Soltec, Milan, Italy) at 60% amplitude for 2 min. The generated nano dispersions were stored in the fridge for later examination.

To prepare SYG-loaded liposomes and oleosomes, the same procedure was followed, and 60 mg SYG was added to the aqueous phase prior to the addition to the thin film.

#### 3.3.2. Preparation of Chitosan Coated Oleosomes (Chito-Oleosomes) (CS-OL)

To prepare CS-OL, the aforementioned procedure was adopted, but the film hydration was conducted using 0.05% *w*/*v* CS solution in 0.5% acetic acid (pH 5.5). Then, the resultant chito-oleosomal dispersion was ultrasonicated at 60% amplitude for 2 min in an ice bath.

#### 3.3.3. Preparation of Folic Acid Functionalized Chito-Oleosomes (F-CS-OL)

Folic acid (0.1% *w*/*v*) was dissolved in a 0.1 N NaOH solution under gentle stirring to obtain a clear solution. CS-OL was mixed under magnetic stirring with an equal volume of 0.1% *w*/*v* folic acid solution for 30 min at RT. The prepared F-CS-OL formulations were washed 3–4 times with deionized water, centrifuged at 16,000 rpm for 20 min, and freeze-dried [19]. The freeze-dried formulations were stored at 4 ± 1 °C in sealed light-resistant containers until further characterization and biological evaluation.

### 3.4. Characterization of OL, CS-OL and F-CS-OL

#### 3.4.1. Particle Size, Polydispersity Index (PDI) and Zeta Potential Assessment

Particle size, PDI and zeta potential were assessed by the Malvern Zeta Sizer Nano ZS from Malvern Instruments (Malvern, UK). Samples were diluted with distilled water in a ratio of 1:50. The samples for particle size analysis were handled in square glass cuvettes, while those for zeta potential were contained in transparent disposable zeta cells. Measurements were performed in triplicate at room temperature. Samples were measured three times.

#### 3.4.2. Morphological Examination

Following uranyl acetate staining, the chosen formulation was morphologically examined using a transmission electron microscope (TEM, JEM-100CX; JEOL, Tokyo, Japan).

#### 3.4.3. Entrapment Efficiency (EE%) and Drug Loading (DL%)

Using a modified centrifugal ultrafiltration method and a Centrisart-I tube (MWCO 300 kDa, Sartorius AG, Goettingen, Germany), the entrapment efficiency (EE) of SYG integrated into oleosomes was evaluated. Two milliliters of the formula were put into the external tube of the Centrisart set. After that, the set was put in a cooling centrifuge (Sigma 3-30KS, Sigma Laborzentrifugen GmbH, Osterode, Germany) for 12 min at 5000 rpm and 4 °C. The interior tube of the Centrisart was used to retain the supernatant. An Agilent Cary 60 UV-Vis Spectrophotometer (Santa Clara, CA, USA) was used to detect the amount of SYG in the supernatant spectrophotometrically at 250 nm in order to create a calibration curve.

$$\mathrm{EE}\%=\frac{\mathrm{Encapsulated}\;\mathrm{SYG}}{\mathrm{Total}\;\mathrm{SYG}}\;\times \;100$$

DL% was calculated using the following equation:

$$\mathrm{DL}\%=\frac{\mathrm{Weight}\;\mathrm{of}\;\mathrm{encapsulated}\;\mathrm{SYG}}{\mathrm{Total}\;\mathrm{wieght}\;\mathrm{of}\;\mathrm{formulation}}\;\times \;100$$

Absorbance measurements were performed at 250 nm using appropriate formulation blanks containing all excipients except SYG extract to correct for background absorbance arising from phospholipids, oleic acid, CS, folic acid, cholesterol, and Tween 80. Thus, SYG quantification was based on the net absorbance after blank subtraction.

#### 3.4.4. In Vitro Drug Release

Dialysis bags (Visking^®^ 36/32, 21 mm, MWCO 12,000–14,000; Serva, Heidelberg, Germany) were used to assess the in vitro drug release. The bags were immersed in distilled water for 24 h and then sealed tightly. SYG solution and the selected F-CS-OL (F7) formulation were accurately weighed, and an amount equivalent to 6 mg SYG was placed in dialysis bags, which were immersed in 50 mL PBS (pH 7.4) as the release medium, following the methodology previously described [67]. The glass bottles were kept at 37 °C and 100 rpm in a water bath shaken thermostatically. A 2 ml sample was collected at predetermined time intervals (0.5, 1, 2, 4, 6, 8 and 24 h) and compensated with the same volume of fresh buffer to ensure sink conditions. This was followed by plotting the average cumulative percentage of SYG released at various time intervals and different drug-release profiles were determined. The drug concentration was assessed by spectrophotometry by measuring the UV absorbance at 250 nm against blank buffer solution.

#### 3.4.5. Fourier Transform Infrared Spectroscopy (FTIR)

FTIR spectra were recorded for SYG, CS, phospholipon 90 H, folic acid, oleic acid and SYG-loaded F-CS-OL by an Agilent Cary 630 FTIR spectrometerSanta Clara, CA USA, at 400–4000 cm^−1^ using an attenuated total reflectance (ATR)-FTIR spectrometer. Samples were analyzed directly using the ATR accessory without potassium bromide (KBr) pellet preparation.

#### 3.4.6. Differential Scanning Calorimetry (DSC)

The thermal properties of the SYG, CS and SYG-loaded F-CS-OL were assessed using differential scanning calorimetry (DSC) (DSC Model 6, Perkin Elmer Instruments, Waltham, MA, USA) with a heating rate of 10 °C/min from 10 °C to 400 °C under nitrogen.

#### 3.4.7. Stability Study

To evaluate the physical stability of the prepared vesicles, the optimized formulation and the corresponding blank formulation were stored in sealed glass vials at 4 ± 1 °C for a period of 3 months. Samples were withdrawn after 1 and 3 months of storage and characterized for particle size, PDI, and zeta potential using dynamic light scattering. In addition, the EE% was determined at each time point using the method described previously. All measurements were performed in triplicate and expressed as mean ± SEM.

### 3.5. In Vivo Study

#### 3.5.1. Animals

Twenty-five adult male Sprague-Dawley rats (180 ± 20 g) were acquired from the animal house of Cairo University’s Faculty of Pharmacy in Cairo, Egypt, and they were acclimated to lab conditions over the course of a week. The animals were kept at 25 ± 2 °C in polyacrylic cages with a 12/12 h light and dark cycle. Regular food and tap water were also freely available to them. Throughout the entire experiment, the ARRIVE guidelines were followed. This experimental study was approved by the Institutional Animal Care and Use Committee (IACUC) of the High Institute of Public Health, Alexandria University, under approval number AU0925123014, and the approval date was 30 December 2025.

#### 3.5.2. Arthritis Induction and Experimental Animals

##### Experimental Design

**Figure 12:**
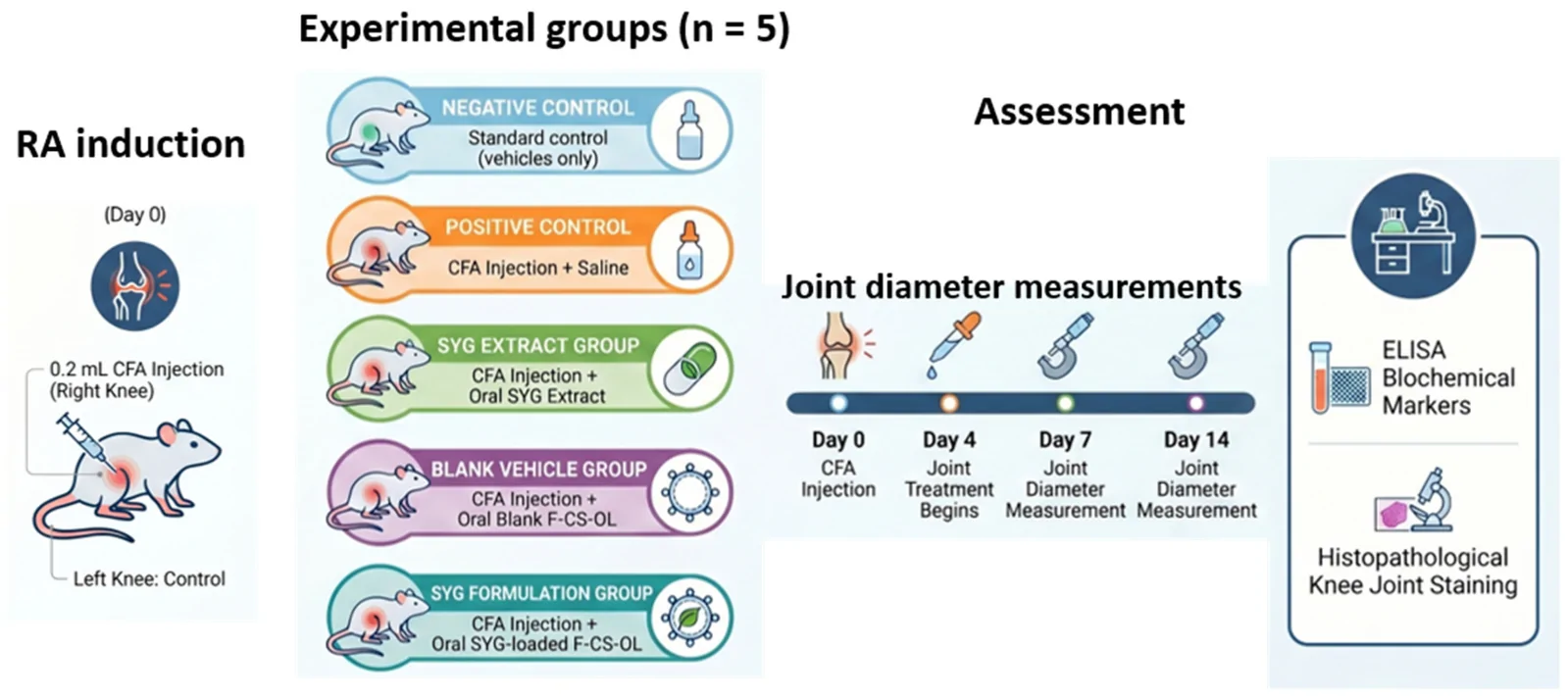
In vivo schematic diagram and experimental groups.

Following acclimatization, animals were randomly assigned to the experimental groups. Five groups (*n* = 5) of twenty-five male rats were randomly selected. The previously published method [52] was used to induce experimental arthritis. In conclusion, a single injection of 0.2 mL of complete Freund’s adjuvant (CFA) into the rats’ right knee joints induced experimental arthritis. CFA was not diluted or emulsified with saline prior to administration. The two control groups were the positive control, in which CFA-arthritis was induced and received no treatment, and the negative control that neither underwent induction nor treatment (normal). Figure 12 shows the experimental groups and the treatments that correlate with them.

The experimental groups were Group I (Negative Control): a standard control group that was given only vehicles. Complete Freund’s Adjuvant (CFA) was used to induce RA in groups II, III, IV, and V. Group II (Positive Control) and Group III (free extract) received a daily dose of 200 mg/kg SYG extract by oral gavage [68]. Rats in Group IV received blank F-CS-OL and SYG-loaded F-CS-OL was administered orally to animals in Group V at a daily dose of (200 mg/kg, and the administered volume was 1 mL in all groups [68]. Treatment initiated on the fourth day of induction and continued till day 14 of the experiment.

All in vivo outcome assessments, including knee joint diameter measurements, biochemical analyses, and histopathological evaluations, were performed by investigators blinded to treatment allocation.

##### Joint Diameter and Arthritis Index Measurements

The anteroposterior rat knee joint diameters were assessed using a micrometer (KM-211-101, Shaanxi, China) on days 0, 4, 7 and 14, and the mean value of three measurements were estimated. Macroscopic evaluation of clinical arthritis progression was performed on days 0, 4, 7, and 14 following disease induction across all experimental groups. The visual severity of joint inflammation, swelling, and erythema was graded on a standard 0-to-4 macroscopic arthritis scale as follows: 0: Normal joint appearance with no detectable erythema or swelling; 1: Mild swelling and/or localized erythema confined to individual digits or small periarticular tissues; 2: Moderate erythema with pronounced swelling affecting the ankle or wrist joint; 3: Severe, widespread swelling and erythema extending across the entire paw; 4: Maximal inflammation accompanied by joint deformity, ankylosis, or total loss of flexibility. The macroscopic arthritis score for each animal was recorded on a 0–4 scale based on the right knee joint. Data are presented as mean ± SEM (*n* = 5 per group).

##### Biochemical Analysis

At the end of the experiment, after an overnight fast (12–14 h), 0.8 mL of blood was drawn from the retro-orbital plexus, where blood is found during anesthesia, followed by 60 mg/kg ketamine and 6 mg/kg xylazine [69]. Blood was centrifuged at 3500 rpm for 10 min for the separation of serum, refrigerated overnight at 4 °C, and stored in 200 µL units at −80 °C for further analysis. The rats were quickly killed by cervical dislocation as soon as the blood was extracted. Commercial ELISA kits were used to measure serum levels of TNF-α, IL-6, CRP, VEGF, ICAM-1, MMP-3, and CXCL13, following manufacturer instructions. Table 5 provides additional information about the origins of these ELISA kits.

##### Histopathological Examination

At the conclusion of the experiment, the animals were slaughtered, and samples of their knee joints were collected. After being fixed in 10% neutral buffer formalin and decalcified in 10% formic acid for 24–48 h, the specimens were trimmed, cleaned in water, dehydrated in increasing ethyl alcohol grades, clarified in xylene, and embedded in paraffin. Hematoxylin and Eosin (H&E), Safranin O, and Toluidine Blue stains were used to prepare and stain a thin segment (4–6µ) [70]. Mankin scoring was used to score tidemark integrity, cells, and structure. For each animal, three sections were prepared and examined. Scoring was performed in a blinded manner by two independent observers. Interobserver reproducibility was assessed and showed high agreement. The number of fields examined for each section was five; random high-power fields were evaluated to ensure representative sampling.

### 3.6. Statistical Analysis

Data are presented as mean ± standard error of the mean (SEM). Biochemical and other single-endpoint measurements were analyzed using one-way ANOVA followed by Tukey’s multiple-comparison post hoc test. Joint diameter measurements, which were recorded longitudinally at multiple time points, were analyzed using two-way ANOVA to evaluate the effects of treatment, time, and their interaction. Statistical analyses were performed using GraphPad Prism version 8.4.2 (GraphPad Software, San Diego, CA, USA).

Five animals were allocated to each experimental group and completed the in vivo study. Joint diameter measurements and histopathological evaluations were performed using all animals (*n* = 5/group). For certain biochemical analyses, four samples per group (*n* = 4) were available because one serum sample from each group provided insufficient volume for the complete analytical panel.

## 4. Conclusions

The -SYG-loaded F-CS-OL formulation consistently and significantly outperformed the free extract treatment across all measured parameters, including inflammation, angiogenesis, adhesion, matrix degradation, and B-cell chemoattraction mediators. The SYG-loaded F-CS-OL group demonstrated the greater therapeutic effects provided by the nano-delivery system by achieving substantial reduction in CRP and IL-6 levels and the largest percent reductions in all other parameters. Although particle size, zeta potential, and FTIR analyses supported association of folic acid with the vesicular system, neither chitosan nor folic acid surface density was quantified. In addition, residual folic acid in the washing supernatants was not analyzed. Therefore, the extent of surface functionalization and the possibility of non-specifically adsorbed folic acid cannot be determined from the current data. Consequently, the enhanced efficacy observed with F-CS-OL cannot be conclusively attributed to folate receptor-mediated targeting alone.

### 4.1. Clinical Implications

The SYG-loaded F-CS-OL formulation outperformed the free extract in all measured efficacy parameters, suggesting the potential value of nano-encapsulation strategies for enhancing the therapeutic activity of SYG in RA. The observed modulation of inflammatory, angiogenic, matrix-degrading, and chemotactic mediators indicates a multi-target pharmacological effect that warrants further investigation. However, comprehensive safety, pharmacokinetic, and biodistribution studies are required before the translational potential of this formulation can be fully established. Moreover, results stem from a single animal model with a single dose and short treatment duration, lacking evidence of clinical efficacy or safety in humans. Before considering clinical translation, future research should focus on dose–response evaluations, long-term safety assessments, biodistribution analyses, and validation in more preclinical models.

### 4.2. Limitations

The total phenolic, flavonoid, tannin, and Vanillin-reactive saponin contents determined in the present study provide an overall estimation of these phytochemical classes but do not permit the identification or quantification of individual constituents. Although previous studies have reported specific bioactive compounds in SYG, these compounds were not individually quantified in the present study. Therefore, targeted chromatographic analysis, such as HPLC-DAD or HPLC-MS, is warranted in future studies to identify and quantify representative phenolic acids, flavonoids, and other bioactive constituents and to further elucidate their contribution to the observed antioxidant and biological activities.

Although particle size, zeta potential, and FTIR analyses support successful folic acid surface functionalization, additional analytical techniques such as X-ray photoelectron spectroscopy (XPS), nuclear magnetic resonance (NMR), quantitative folate determination, or dedicated conjugation assays would be required to unequivocally confirm covalent attachment and quantify surface-bound folate.

The present study did not include a standard DMARD-treated positive control group, such as methotrexate or leflunomide, and therefore direct comparison with established anti-rheumatic therapies could not be made. In addition, only a single dose level of SYG (200 mg/kg) was evaluated, precluding assessment of dose–response relationships and determination of the minimum effective dose. Future studies should incorporate clinically relevant DMARD comparators and multiple dosing levels to better define the therapeutic positioning and translational potential of the developed nanoformulation.

While the present study was designed to evaluate therapeutic efficacy rather than cellular uptake mechanisms, the choice of folic acid as a targeting moiety was supported by extensive literature demonstrating FRβ overexpression on activated macrophages in RA. We acknowledge that dedicated receptor-blocking, cellular uptake, or biodistribution studies would provide more direct evidence of FRβ-mediated targeting and will be considered in future investigations.

Although the developed F-CS-OL system demonstrated favorable physicochemical characteristics and improved therapeutic efficacy in vivo, the present study did not directly investigate pharmacokinetic behavior, systemic exposure, biodistribution, or oral bioavailability of SYG constituents. Therefore, any potential enhancement in circulation time, tissue accumulation, or bioavailability remains hypothetical and warrants confirmation through dedicated pharmacokinetic and biodistribution studies.

Nevertheless, the present study did not include a comprehensive systemic safety assessment. Parameters such as body weight changes, food consumption, organ weights, hematological indices, serum biochemical markers, and histopathological examination of major organs were not evaluated. Therefore, the safety profile of the developed formulation remains to be established through dedicated toxicological studies. In addition, pharmacokinetic and biodistribution studies are required to characterize systemic exposure and potential off-target accumulation before clinical translation can be considered.

Finally, A formal a priori sample-size or statistical power calculation was not performed prior to study initiation. The group size was selected based on previous studies utilizing similar experimental arthritis models and outcome measures. Although significant differences were detected for multiple endpoints, the relatively small number of animals per group may limit statistical power for some analyses. Therefore, the findings should be interpreted as preliminary preclinical evidence and should be confirmed in larger, adequately powered studies.

## Figures and Tables

**Table 1:** Phytochemical and functional characterization of SYG seed extract.

SYG Seed Extract	Mean ± SE (*n* = 3)
Total vanillin-reactive saponin (mg AE/gm dry extract)	10.38 ± 0.1
Total Tannins mg (Tannic Equivalent)/gm dry extract)	35.53 ± 0.1
Total phenolic (con. mg (GAE)/gm dry extract)	40.49 ± 0.0
Total flavonoid con. mg (QE)/gm dry extract)	30.49 ± 0.0

**Table 2:** Composition and colloidal characteristics of different formulations.

Formulation	SYG	Oleic Acid	CS	Folic Acid	Mean Diameter (nm)	PDI	Zeta Potential (mV)	EE%	Drug Loading (DL%)
F1	-------	-------	-------	------	54.39 ± 0.56	0.182 ± 0.002	−16.2 ± 1.44	------	------
F2	-------	50 mg	-------	------	47.41 ± 0.87	0.180 ± 0.001	−20.6 ± 1.32	------	------
F3	60 mg		------	------	54.42 ± 0.34	0.180 ± 0.020	−15.1 ± 0.98	91.45 ± 1.2	15.59 ± 0.20
F4	------		0.05 % *w*/*v*	------	60.15 ± 0.34	0.187 ± 0.004	16.7 ± 2.1	------	------
F5	60 mg			------	66.80 ± 0.98	0.250 ± 0.025	17.25 ± 0.56	94.78 ± 0.92	16.16 ± 0.16
F6	------			0.1% *w*/*v*	61.60 ± 0.34	0.263 ± 0.013	−14.7 ± 1.89	------	------
F7	60 mg				80.78 ± 1.02	0.227 ± 0.012	−15.5 ± 1.12	94.98 ± 0.56	16.19 ± 0.10

Cholesterol (2 mg) and Tween 80 (40 mg) were included in all formulations in the same concentrations.

**Table 3:** Stability data of F6 and F7 after storage for 1 and 3 months at 4 °C.

Formulation	Fresh		1 Month		3 Months	
	F6	F7	F6	F7	F6	F7
Mean diameter (nm)	61.60 ± 0.34	80.78 ± 1.02	62.22 ± 0.23	81.56 ± 0.87	63.67 ± 1.03	83.41 ± 0.58
PDI	0.263 ± 0.013	0.227 ± 0.012	0.273 ± 0.01	0.230 ± 0.02	0.279 ± 0.01	0.232 ± 0.03
Zeta potential (mV)	−14.7 ± 1.89	−15.5 ± 1.12	−14.5 ± 1.53	−15.2 ± 1.32	−14.3 ± 1.28	−15.1 ± 1.56
EE%	------	94.98 ± 0.56	------	93.87 ± 0.34	------	93.34 ± 0.76

**Table 4:** Histopathological scoring in different experimental groups.

Comparison Criteria	Negative Control	Positive Control	SYG Solution	Blank F-CS-OL	SYG-Loaded F-CS-OL
Structure	0	4	1	1	0
Cells	0	3	2	3	0
Tidemark integrity	0	4	3	2	0
Staining	0	4	3	3	0
Total Mankin score	0 ± 0	15 ± 1.2	9 ± 1.2	9 ± 1.5	0 ± 0

**Table 5:** ELISA kits used in the study.

Protein	Manufacturer and Catalog No.	Species	Assay Range
TNF-α	Abcam TNF alpha ELISA Kit, Catalog No: ab236712	Rat	15.6–1000 pg/mL
IL-6	Elab science IL-6 ELISA Kit, Catalog No: E-EL-R0015	Rat	31.25–2000 pg/mL
CRP	My BioSource hs-CRP ELISA Kit, Catalog No: MBS2508830	Rat	0.156–10 ng/ml
ICAM-1	Elab science ICAM-1/CD54 ELISA Kit, Catalog No: E-EL-R2850	Rat	0.31–20 ng/mL
VEGF	My BioSource VEGF Elisa kit, Catalog No: MBS724516	Rat	15.6–1000 pg/mL
MMP3	Elab science^®^ MMP-3 ELISA Kit, Catalog No: E-EL-R0619	Rat	0.156–10 ng/mL
CXCL13	My BioSource CXCL13/BLC/BCA-1 Elisa kit, Catalog No: MBS2601738	Rat	31.25–2000 pg/mL

## Data Availability

The original contributions presented in this study are included in the article/Supplementary Materials. Further inquiries can be directed to the corresponding author.

## Supplementary Materials

The following supporting information can be downloaded at: https://www.mdpi.com/article/10.3390/ph19101658/s1, Figure S1: UV spectrophotometry absorbance spectra for SYG seed extract in different concentrations ranging from (10–40 µg/mL).

## References

[1] Zhao F., Hu Z., Li G., Liu M., Huang Q., Ai K., Cai X. (2025). Angiogenesis in rheumatoid arthritis: Pathological characterization, pathogenic mechanisms, and nano-targeted therapeutic strategies. Bioact. Mater..

[2] Pulik Ł., Łęgosz P., Motyl G. (2023). Matrix metalloproteinases in rheumatoid arthritis and osteoarthritis: A state of the art review. Reumatologia.

[3] ELhabal S.F., El-Nabarawi M.A., Hassanin S.O., Hassan F.E., Abbas S.S., Gebril S.M., Albash R. (2025). Transdermal fluocinolone acetonide loaded decorated hyalurosomes cellulose acetate/polycaprolactone nanofibers mitigated Freund’s adjuvant-induced rheumatoid arthritis in rats. J. Pharm. Investig..

[4] Smith M.H., Gao V.R., Periyakoil P.K., Kochen A., DiCarlo E.F., Goodman S.M., Norman T.M., Donlin L.T., Leslie C.S., Rudensky A.Y. (2023). Drivers of heterogeneity in synovial fibroblasts in rheumatoid arthritis. Nat. Immunol..

[5] Chen Y., Han T., Guo Z., Li X., Zhang X., Peng W. (2024). Immunomodulatory Roles of Fibroblast-like Synoviocytes in Rheumatoid Arthritis. Front. Immunol..

[6] Bian Y., Xiang Z., Wang Y., Ren Q., Chen G., Xiang B., Wang J., Zhang C., Pei S., Guo S. (2023). Immunomodulatory roles of metalloproteinases in rheumatoid arthritis. Front. Pharmacol..

[7] Liu L., Li X., Wu Z., Yang L., Huo J., Tang S., Cao X., Xu Y., Liao X., Qi H. (2024). Inflammation-targeting fullerene nanoparticles dually inhibit macrophage and osteoclast differentiation for mitigating rheumatoid arthritis. CCS Chem..

[8] Chen W., Fang Y., Wang H., Tan X., Zhu X., Xu Z., Jiang H., Wu X., Hong W., Wang X. (2023). Role of chemokine receptor 2 in rheumatoid arthritis: A research update. Int. Immunopharmacol..

[9] Wang B., Wang M., Ao D., Wei X. (2022). CXCL13-CXCR5 axis: Regulation in inflammatory diseases and cancer. Biochim. Biophys. Acta (BBA)-Rev. Cancer.

[10] Smolen J.S., Landewé R.B., Bergstra S.A., Kerschbaumer A., Sepriano A., Aletaha D., Caporali R., Edwards C.J., Hyrich K.L., Pope J.E. (2023). EULAR recommendations for the management of rheumatoid arthritis with synthetic and biological disease-modifying antirheumatic drugs: 2022 update. Ann. Rheum. Dis..

[11] Fraenkel L., Bathon J.M., England B.R., St. Clair E.W., Arayssi T., Carandang K., Deane K.D., Genovese M., Huston K.K., Kerr G. (2021). 2021 American College of Rheumatology guideline for the treatment of rheumatoid arthritis. Arthritis Rheumatol..

[12] Szekanecz Z., Buch M.H., Charles-Schoeman C., Galloway J., Karpouzas G.A., Kristensen L.E., Ytterberg S.R., Hamar A., Fleischmann R. (2024). Efficacy and safety of JAK inhibitors in rheumatoid arthritis: Update for the practising clinician. Nat. Rev. Rheumatol..

[13] Ayyanar M., Subash-Babu P. (2012). Syzygium cumini (L.) Skeels: A review of its phytochemical constituents and traditional uses. Asian Pac. J. Trop. Biomed..

[14] Kumar V., Khatri N., Kumar D., Shekhawat M., Vandana Garg V., Kumar A., Kakkar S. (2025). Modern perspectives on the traditional uses, phytochemical profiles, and therapeutic benefits of Syzygium cumini. Integr. Med. Discov..

[15] Zewail M., Abbas H., El Sayed N., El Newehy N.M., Abd-El-Azim H. (2025). Intradermal Delivery of Nanohybrid Thermosensitive Hydrogels Loaded with Chondroitin Sulphate Coated Pomegranate Lipid Carriers Via Hollow Microneedles for Effective Rheumatoid Arthritis Management. J. Drug Deliv. Sci. Technol..

[16] El-Nashar H.A., Abbas H., Zewail M., Noureldin M.H., Ali M.M., Shamaa M.M., Khattab M.A., Ibrahim N. (2022). Neuroprotective Effect of Artichoke-Based Nanoformulation in Sporadic Alzheimer’s Disease Mouse Model: Focus on Antioxidant, Anti-Inflammatory, and Amyloidogenic Pathways. Pharmaceuticals.

[17] Makhlof M.E., Zewail M., Abo-Neima S.E., Alfaifi M.Y., Shati A.A., Mokhtar F.A. (2026). Biophysical Characterization of Wafers Co-loading Quercetin Loaded Chitosan-Coated Oleosomes and Polycladia Myrica-Mediated Zinc Oxide Nanoparticles for Antiviral, Anti-inflammatory and Hepatoprotective Therapy. J. Polym. Environ..

[18] Sandhya S., Lokapur J.S., Goudanavar P.S. (2026). A Comprehensive Review on Oleic Acid Vesicles: A Novel Approach to Drug Delivery. Pharm. Nanotechnol..

[19] Zewail M. (2021). Folic acid decorated chitosan-coated solid lipid nanoparticles for the oral treatment of rheumatoid arthritis. Ther. Deliv..

[20] Steinz M.M., Ezdoglian A., Khodadust F., Molthoff C.F., Srinivasarao M., Low P.S., Zwezerijnen G.J., Yaqub M., Beaino W., Windhorst A.D. (2022). Folate receptor beta for macrophage imaging in rheumatoid arthritis. Front. Immunol..

[21] Qi R., Majoros I., Misra A.C., Koch A.E., Campbell P., Marotte H., Bergin I.L., Cao Z., Goonewardena S., Morry J. (2015). Folate receptor-targeted dendrimer-methotrexate conjugate for inflammatory arthritis. J. Biomed. Nanotechnol..

[22] Li X., Hou Y., Meng X., Li G., Xu F., Teng L., Sun F., Li Y. (2021). Folate receptor-targeting mesoporous silica-coated gold nanorod nanoparticles for the synergistic photothermal therapy and chemotherapy of rheumatoid arthritis. RSC Adv..

[23] Zhao J., Zhao M., Yu C., Zhang X., Liu J., Cheng X., Lee R.J., Sun F., Teng L., Li Y. (2017). Multifunctional folate receptor-targeting and pH-responsive nanocarriers loaded with methotrexate for treatment of rheumatoid arthritis. Int. J. Nanomed..

[24] Minnady M., Jayapal G., Poochi S., Nethaji P., Mathalaimuthu B. (2022). Hepatoprotective Effect of Indigenous Medicinal Plants: A Review. Indian J. Pharm. Sci..

[25] Zanzabil K.Z., Hossain M.S., Hasan M.K. (2023). Diabetes mellitus management: An extensive review of 37 medicinal plants. Diabetology.

[26] Srivastava S., Chandra D. (2013). Pharmacological potentials of Syzygium cumini: A review. J. Sci. Food Agric..

[27] Agidew M.G. (2022). Phytochemical analysis of some selected traditional medicinal plants in Ethiopia. Bull. Natl. Res. Cent..

[28] Ahmed S., Jahan I.A., Hossain M.H., Ahmed K.S., Rahman M., Zzaman W., Hoque M.M. (2021). Bioactive compounds, antioxidant properties and phenolic profile of pulp and seed of Syzygium cumini. J. Food Meas. Charact..

[29] Imran A., Ye S., Li J.A., Ajaj R., Rauf A., Ahmad Z., Hemeg H.A., Al-Awthan Y.S.M., Bahattab O.S., Quradha M.M. (2025). LC-ESI-QTOF-MS/MS Characterization of Phenolic Compounds in the Stem, Roots, and Leaves of Syzygium cumini and Their Antioxidant Potential. Food Sci. Nutr..

[30] Lombardo D., Kiselev M.A. (2022). Methods of liposomes preparation: Formation and control factors of versatile nanocarriers for biomedical and nanomedicine application. Pharmaceutics.

[31] Singh R.P., Gangadharappa H., Mruthunjaya K. (2017). Phospholipids: Unique carriers for drug delivery systems. J. Drug Deliv. Sci. Technol..

[32] Elhabal S.F., Al-Zuhairy S.A.-K.S., Elrefai M.F.M., El-Nabarawi M.A., Hababeh S., Zarif Attalla K., Shoela M.S., Nelson J., Fady M., Elzohairy N.A. (2025). Chitosan-based intelligent microneedles for delivery of amphotericin B loaded oleosomes: Antifungal ocular patch targeting for effective against fungal keratitis using rabbit model via TLR4/NLRP3 pathway. Int. J. Nanomed..

[33] Talló K., Moner V., De Cabo M., Cócera M., López O. (2018). Vesicular nanostructures composed of oleic acid and phosphatidylcholine: Effect of pH and molar ratio. Chem. Phys. Lipids.

[34] Elnaggar Y.S., Zewail M., Salem E.M., Alghonemy W.Y., Ahmed N.M., Hanafy R.A., Daghistan W., Alaseem A.M., Khodeer D., Zaki E.G. (2026). Tailored Phytochitosomes as Targeted Nanotherapy for Alveolar Bone Regeneration in Diabetic Obese Rats. Pharmaceuticals.

[35] Sebaaly C., Trifan A., Sieniawska E., Greige-Gerges H. (2021). Chitosan-coating effect on the characteristics of liposomes: A focus on bioactive compounds and essential oils: A review. Processes.

[36] Metwaly A.M., Abd-El-Azim H., Zewail M., Alsfouk A.A., Elkaeed E.B., Eissa I.H. (2025). Chitosomal encapsulation enhances the anticancer efficacy of a theobromine analogue: An integrated in silico and in vitro study. J. Comput. Biophys. Chem..

[37] Metwaly A.M., Abd-El-Azim H., Zewail M., Alsfouk A.A., Elkaeed E.B., Elkady H., Eissa I.H. (2025). Exploring the Anticancer Potential of Chitosome-Encapsulated Theobromine Analogue: Integrated Computational, Physiochemical and In vitro Study. Nat. Prod. Commun..

[38] Nematalla H.A., Elharoun M., Abd-Alhaseeb M.M., Sharafeldin H.A., Elsheikh M.A., Abbas H., Elkelish A., Dossouvi K.M., Mehana A.E., Abdelgawad F.E. (2025). Innovative approach in Parkinson’s targeting via berberine-loaded mucoadhesive surface-modified liposomes: A multi-faceted study. BMC Pharmacol. Toxicol..

[39] Nematalla H.A., Elharoun M., Bargash S.N., Abd-Alhaseeb M.M., Sharafeldin H.A., Zewail M., Abbas H., Elsheikh M.A. (2025). Novel Nose-to-brain delivery of carbenoxolone via mucoadhesive solid lipid nanoparticles for Parkinson’s symptoms management: In vitro and in vivo evaluation in a rotenone-induced rat model. Int. J. Pharm..

[40] Nguyen C.N., Tran B.N., Thi H.N., Huu P.P., Thi H.N. (2019). Physical Absorption of Folic Acid and Chitosan on Dihydroartemisinin-Loaded Poly-Lactic-Co-Glycolic Acid Nanoparticles via Electrostatic Interaction for Their Enhanced Uptake and Anticancer Effect. J. Nanomater..

[41] Abdelalim L.R., Elnaggar Y.S., Abdallah O.Y. (2020). Oleosomes encapsulating sildenafil citrate as potential topical nanotherapy for palmar plantar erythrodysesthesia with high ex vivo permeation and deposition. AAPS PharmSciTech.

[42] Elhalmoushy P.M., Elsheikh M.A., Matar N.A., El-Hadidy W.F., Kamel M.A., Omran G.A., Elnaggar Y.S. (2023). Elaboration of novel gel-core oleosomes encapsulating phytoconstituent for targeted topical delivery in a vitiligo-induced mouse model: Focus on antioxidant and anti-inflammatory pathways. J. Drug Deliv. Sci. Technol..

[43] Zewail M., Nafee N., Helmy M.W., Boraie N. (2019). Coated nanostructured lipid carriers targeting the joints–an effective and safe approach for the oral management of rheumatoid arthritis. Int. J. Pharm..

[44] Kang B.R., Park J.S., Ryu G.R., Jung W.-J., Choi J.-S., Shin H.-M. (2022). Effect of chitosan coating for efficient encapsulation and improved stability under loading preparation and storage conditions of bacillus lipopeptides. Nanomaterials.

[45] Alcantara K.P., Pajimna R.M.B., Aliga P.J.S., Malabanan J.W.T., Tangwongsiri C., Haworth I.S., Rojsitthisak P., Rojsitthisak P. (2025). Review of Chitosan-Coated Nanoscale Liposomes for Enhanced Drug Delivery. ACS Appl. Nano Mater..

[46] Zhuang J., Ping Q., Song Y., Qi J., Cui Z. (2010). Effects of chitosan coating on physical properties and pharmacokinetic behavior of mitoxantrone liposomes. Int. J. Nanomed..

[47] Thiagamani S.M.K., Yaswanth C.V., Yashwanth C., Tran T.M.N., Krishnasamy S., Azhaguchamy M., Khan A., Hashem M., Fouad H. (2024). Characterization of Syzygium cumini (L.) Skeels (Jamun seed) particulate fillers for their potential use in Polymer composites. Molecules.

[48] Saoji S.D., Raut N.A., Dhore P.W., Borkar C.D., Popielarczyk M., Dave V.S. (2016). Preparation and evaluation of phospholipid-based complex of standardized centella extract (SCE) for the enhanced delivery of phytoconstituents. AAPS J..

[49] Tiwari A., Singh A., Debnath A., Kaul A., Garg N., Mathur R., Singh A., Randhawa J.K. (2019). Multifunctional magneto-fluorescent nanocarriers for dual mode imaging and targeted drug delivery. ACS Appl. Nano Mater..

[50] Muñoz-Núñez C., Gómez-Fernández N., Muñoz-Bonilla A., Fernández-García M. (2025). Chemical Modification of Chitosan with Bioactive Molecules: A Sustainable Approach for Advanced Film Development. Int. J. Mol. Sci..

[51] Ferrero F., Periolatto M. (2012). Antimicrobial finish of textiles by chitosan UV-curing. J. Nanosci. Nanotechnol..

[52] Zewail M., El-Deeb N.M., Mousa M.R., Abbas H. (2022). Hyaluronic acid coated teriflunomide (A771726) loaded lipid carriers for the oral management of rheumatoid arthritis. Int. J. Pharm..

[53] Murayama M.A., Shimizu J., Miyabe C., Yudo K., Miyabe Y. (2023). Chemokines and chemokine receptors as promising targets in rheumatoid arthritis. Front. Immunol..

[54] Salem H.F., Nafady M.M., Kharshoum R.M., Abd El-Ghafar O.A., Farouk H.O. (2020). Mitigation of rheumatic arthritis in a rat model via transdermal delivery of dapoxetine HCl amalgamated as a nanoplatform: In vitro and in vivo assessment. Int. J. Nanomed..

[55] Ciurtin C., Helmy G.A., Ferreira A.C., Manson J.J., Jury E.C., McDonnell T. (2024). A tale of two functions: C-reactive protein complementary structures and their role in rheumatoid arthritis. Clin. Immunol..

[56] Alunno A., Carubbi F., Giacomelli R., Gerli R. (2017). Cytokines in the pathogenesis of rheumatoid arthritis: New players and therapeutic targets. BMC Rheumatol..

[57] Pope J.E., Choy E.H. (2021). C-Reactive Protein and Implications in Rheumatoid Arthritis and Associated Comorbidities.

[58] Zhan H., Li H., Liu C., Cheng L., Yan S., Li Y. (2021). Association of circulating vascular endothelial growth factor levels with autoimmune diseases: A systematic review and meta-analysis. Front. Immunol..

[59] Achudhan D., Lai Y.-L., Lin Y.-Y., Huang Y.-L., Tsai C.-H., Ho T.-L., Ko C.-Y., Fong Y.-C., Huang C.-C., Tang C.-H. (2024). CXCL13 promotes TNF-α synthesis in rheumatoid arthritis through activating ERK/p38 pathway and inhibiting miR-330-3p generation. Biochem. Pharmacol..

[60] Sun S., Sun Y., Shen J., Mao Z., Zhang T., Gao Y., Yang D., Chen X., Song G. (2025). CXCL13/CXCR5: A new target for pain treatment. Int. J. Surg..

[61] Elnaggar Y.S., Zewail M., Salem E.M., Alghonemy W.Y., Ahmed N.M., Abdelfatah O.M., Hanafy R.A., Moustafa M.A. (2026). Development and Appraisal of Luteolin-Loaded Nanohyaluosomes as a Sustainable Tissue Engineering Nanotherapy for Treatment of Periodontitis: Biochemical and Biological Investigations. J. Drug Deliv. Sci. Technol..

[62] Mohanty S., Konkimalla V.B., Pal A., Sharma T., Si S.C. (2021). Naringin as sustained delivery nanoparticles ameliorates the anti-inflammatory activity in a Freund’s complete adjuvant-induced arthritis model. ACS Omega.

[63] Le A.V., Parks S.E., Nguyen M.H., Roach P.D. (2018). Improving the vanillin-sulphuric acid method for quantifying total saponins. Technologies.

[64] Bajaj K., Devsharma A. (1977). A colorimetric method for the determination of tannins in tea. Microchim. Acta.

[65] Singleton V.L., Orthofer R., Lamuela-Raventós R.M. (1999). [14] Analysis of total phenols and other oxidation substrates and antioxidants by means of folin-ciocalteu reagent. Methods in Enzymology.

[66] Re R., Pellegrini N., Proteggente A., Pannala A., Yang M., Rice-Evans C. (1999). Antioxidant activity applying an improved ABTS radical cation decolorization assay. Free Radic. Biol. Med..

[67] Ayyanar C.B., Bal T., Sharma S., Gayathri B., Rinusuba V., Nalini H.E., Deepa S., Dharshinii M.D., Kharra P., Sinha A. (2024). In-vitro and in-vivo investigation of wound healing efficacy of Syzygium cumini leaf extracts loaded carboxymethylcellulose film. Int. J. Biol. Macromol..

[68] Sutrisno S., Prayitno A., Wijayanti L., Nurudhin A., Nurwati I., Pamungkasari E.P. (2025). Effectiveness of Juwet Fruit (Syzygium cumini) in Lowering C-Reactive Protein (CRP), Rheumatoid Factor (RF), and Malondialdehyde (MDA) Levels in Rheumatoid Arthritis-Induced Wistar Rats. Trop. J. Nat. Prod. Res..

[69] Sotoudeh N., Namavar M.R. (2022). Optimisation of ketamine-xylazine anaesthetic dose and its association with changes in the dendritic spine of CA1 hippocampus in the young and old male and female Wistar rats. Vet. Med. Sci..

[70] Bancroft J.D., Gamble M. (2008). Theory and Practice of Histological Techniques.

