# Supplementary material for: Folic Acid-Functionalized Chitosan-Coated Oleosomes Engineered for Enhanced Anti-Arthritic Delivery of Syzygium cumini Seed Extract
Source: Pharmaceuticals (Basel). 2026 Oct 20;19(10):1658. doi: 10.3390/ph19101658 (PMC13708674; doi:10.3390/ph19101658)
Supplement: Supplementary file 1 [file pharmaceuticals-19-01658-s001.zip › pharmaceuticals-4598944-supplementary.pdf]

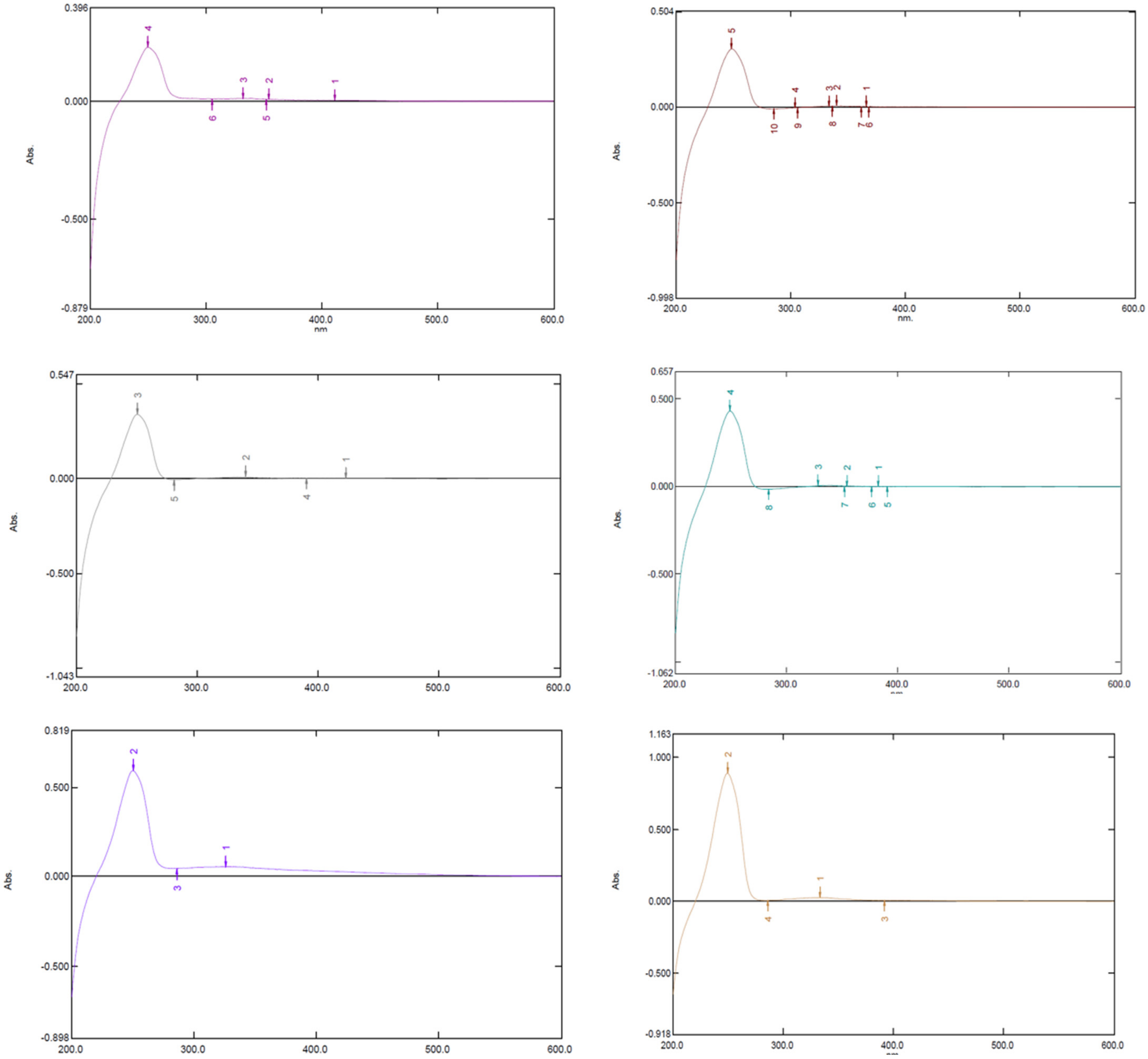

Figure S1: UV spectrophotometry absorbance spectra for SYG seed extract in different concentrations ranging from (10 – 40 µg/ml).
